# Review of Shikonin and Derivatives: Isolation, Chemistry, Biosynthesis, Pharmacology and Toxicology

**DOI:** 10.3389/fphar.2022.905755

**Published:** 2022-07-01

**Authors:** Snehlata Yadav, Ajay Sharma, Gulzar Ahmad Nayik, Raymond Cooper, Garima Bhardwaj, Harvinder Singh Sohal, Vishal Mutreja, Ramandeep Kaur, Franklin Ore Areche, Mohannad AlOudat, Ayaz Mukarram Shaikh, Béla Kovács, Abdelhakam Esmaeil Mohamed Ahmed

**Affiliations:** ^1^ Department of Chemistry, Chandigarh University, Mohali, India; ^2^ University Centre for Research and Development, Department of Chemistry, Chandigarh University, Chandigarh- Ludhiana Highway, Mohali, India; ^3^ Department of Food Science & Technology, Govt. Degree College Shopian, Srinagar, India; ^4^ Department of Applied Biology and Chemical Technology, The Hong Kong Polytechnic University, Kowloon, Hong Kong SAR, China; ^5^ Department of Chemistry, Sant Longowal Institute of Engineering and Technology, Longowal, Sangrur, India; ^6^ Department of Chemistry, Punjab Agricultural University, Ludhiana, India; ^7^ Professional School of Agroindustrial Engineering, National University of Huancavelica, Huancavelica, Peru; ^8^ Doctoral School of Food Science, Hungarian University of Agriculture and Life Sciences, Budapset, Hungary; ^9^ Institute of Food Science, University of Debrecen, Debrecen, Hungary; ^10^ Faculty of Forestry, University of Khartoum, Khartoum North, Sudan

**Keywords:** medicinal plant, shikonin, secondary metabolites, biosynthesis, chemical synthesis, pharmacology, toxicology

## Abstract

Shikonin and its derivatives, isolated from traditional medicinal plant species of the genus *Lithospermum, Alkanna, Arnebia, Anchusa, Onosma, and Echium* belonging to the Boraginaceae family, have numerous applications in foods, cosmetics, and textiles. Shikonin, a potent bioactive red pigment, has been used in traditional medicinal systems to cure various ailments and is well known for its diverse pharmacological potential such as anticancer, antithrombotic, neuroprotective, antidiabetic, antiviral, anti-inflammatory, anti-gonadotropic, antioxidants, antimicrobial and insecticidal. Herein, updated research on the natural sources, pharmacology, toxicity studies, and various patents filed worldwide related to shikonin and approaches to shikonin’s biogenic and chemical synthesis are reviewed. Furthermore, recent studies to establish reliable production systems to meet market demand, functional identification, and future clinical development of shikonin and its derivatives against various diseases are presented.

## 1 Introduction

Shikonin, effective against numerous diseases with negligible side effects, is obtained from dried roots (commonly called zicao) of *Lithospermum erythrorhizon Siebold & Zucc.* It is frequently used as a traditional and modern herbal medicine in China (Chen et al., 2002). A small number of medicinal plants found in the northwestern Himalayas also produce shikonin in the roots (Kumar et al., 2014). Acetylshikonin was the first compound discovered from the roots of *L. erythrorhizon Siebold & Zucc*. Other related species in the Boraginaceae family also contain shikonin derivatives: *Echium lycoris, Arnebia euchroma (Royle) Johnst., Onosma armeniacum K.* (Boraginaceae), *Eritrichium sericeum Lehm.*, *Arnebia Decumbens (Ventenat) Cosson and Kralik*, *Arnebia hispidissima (Lehm.), Lithospermum canescens* (Michx.) *Lehm., Alkanna tinctoria (L.) Tausch, Jatropha glandulifera Roxb.* and *Lithospermum officinale L.* (Saradha Devi et al., 2016; Fu et al., 2020).

**FIGURE 1:**
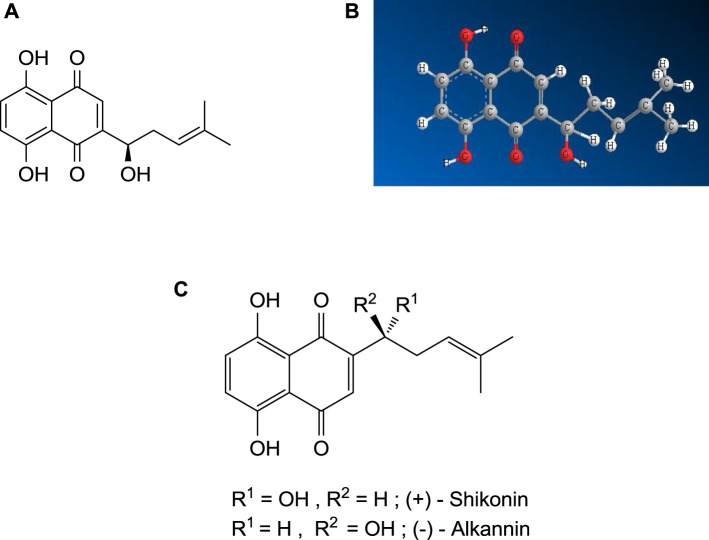
**(A)** Shikonin - 2*D* structure, **(B)** Shikonin — 3*D* structure, **(C)** Shikalkin (a racemic mixture of alkannin and shikonin).

Shikonin is a derivative of 1,4-naphthoquinone (Kumar et al., 2014), biosynthesized from two precursors, geranyl diphosphate (GPP), *via* the mevalonate pathway (Newman and Chappell, 1999) and p-hydroxybenzoic acid (PHB), *via* the phenylpropanoid pathway (Newman and Chappell, 1999). The IUPAC name of shikonin (C_16_H_16_O_5_, shown in Figure 1) is 5, 8-dihydroxy-2-[(1R)-1-hydroxy-4-methyl-3-pentenyl]-1,4-naphthoquinone, determined by Brockmann and Liebigs in 1936, who further determined the enantiomer of shikonin, named alkannin (Saradha Devi et al., 2016). The shikonin (*R*- enantiomer) exists as an enantiomeric pair with alkannin (S- enantiomer), hence known as A/S and shows various pharmacological activities. The chiral pairs A/S are mainly obtained from the roots of around a hundred and fifty species belonging to *Lithospermum, Alkanna, Onosma, Echium, Cynoglossum,* and *Anchusa* of the family Boraginaceae. It was observed that both A and S are synthesized simultaneously during the biogenesis in the identical plant (Papageorgiou et al., 2006). The chiral pairs A/S are well known for their wide range of pharmacological potentials, such as antimicrobial, anticancer, antioxidant, wound healing, anti-inflammatory, and antithrombotic. However, it is very significant to note that both S and A showed almost similar pharmacological properties, although enantiomers of chiral drugs vary substantially in their toxicological and pharmacological properties since these interact with biological macromolecules and the most of them are stereoselective (Papageorgiou et al., 2006).

Further, it is also important to note that the different derivatives of A and S display opposite pharmacological potential, for instance, antimicrobial, anticancer, and wound healing potential, categorizing them as a scarce pair of enantiomeric secondary metabolites (Papageorgiou et al., 2006). Many research work (related to isolation, synthesis, biotechnology, pharmacology and toxicity) has been published on both the A/S chiral pair and their derivative. So, it is hard to compile all the work related to A/S chiral pair in one manuscript; presently, we are only focusing on the shikonin (*R*- enantiomer).

Over the last four decades, shikonin and its derivatives have demonstrated antimicrobial, antioxidant, anti-inflammatory, wound healing, anticancer, antiulcer, anti-angiogenic, and granulated tissue-forming activity (Zhang et al., 2020; Guo et al., 2019; Liu et al., 2020). Some injuries like burns, cuts, and haemorrhoids are also cured by shikonin derivatives (Zhang et al., 2020). Shikonin derivatives possess a cyclopropane moiety and show potential activity against various melanoma cell lines (Kretschmer et al., 2021). Shikonin has shown promise for postmenopausal osteoporosis in reducing bone loss (Chen et al., 2020). Various studies show that shikonin and other derivatives possess potential anti-tumour effects (Shen et al., 2020). Furthermore, it hinders the epidermal growth factor receptor, which signals for human epidermoid carcinoma cells causing cell death (Saradha Devi et al., 2016). Research related to shikonin has demonstrated its several beneficial commercial properties, such as pH-sensing-color changing properties used in food packaging (Roy and Rhim, 2021) and checking the freshness of meat (Ezati et al., 2021a). Although shikonin is considered safe, not enough toxicity is observed; still, various dose-dependent studies of the shikonin derivatives on tissues and cells are yet to be performed (Su et al., 2014).

Shikonin has been used for a long time on a commercial scale and has a high market value. Unfortunately, to meet large-scale market demands, shikonin-producing plants have been overexploited, and as a result, these plants are critically endangered (Kumar et al., 2014). This overexploitation of shikonin-producing plants has led to various advances and efficient chemical synthesis of shikonin and its derivatives. Previously several reviews on shikonin have been published. Wang et al. (2012b) elaborated on the studies on the synthesis and pharmacology of shikonin and its derivatives related to the anticancer activity.

Similarly, Wang et al. (2019) reviewed and presented the mechanism and importance of shikonin in cancer therapy and discussed the various anti-cancer mechanisms of shikonin. Further, Andújar et al. (2013) explained shikonin’s various pharmacological properties, including anticancer, antioxidant, neuroprotective, antimicrobial, cardioprotective, and wound healing as well as glucose metabolism. These reviews also presented the biosynthesis and chemical synthesis of shikonin. The most recent review on shikonin was related to its pharmacology, pharmacokinetics, toxicology, clinical trials, and pharmaceutical applications, presenting all the aspects of shikonin in detail as mentioned above (Sun et al., 2022). In comparison to these articles, the present review includes all the possible aspects of shikonin and its derivatives, including various natural sources, distribution, isolation, biosynthesis, chemical synthesis, stability, pharmacology, patent, and toxicity. Including every aspect of shikonin compared to previous review articles and future prospective offers new advances compared to previously available reviews.

## 2 Materials and Methods

### 2.1 Strategy

The PRISMA (Preferred reporting of items for systematic reviews and meta-analysis) method was applied for the current review to check the data collected, included, and excluded to complete the study. The extensive literature survey from Science Direct, Wiley online library, ACS, Springer, Research gate, Google Scholar, Google Patents, and ClinicalTrials.gov (https://clinicaltrials.gov/) was undertaken by the authors. The keyword combinations were used such as “shikonin source,” “shikonin derivatives,” “shikonin pharmacology,” “shikonin toxicology,” and “shikonin biosynthesis” and “shikonin chemical synthesis”. The research articles included in this review follow a specific standard of scientific criteria. The authors identified 294 articles from databases and other sources. From this list, 78 articles were excluded because of duplication and repetitions.

**FIGURE 2:**
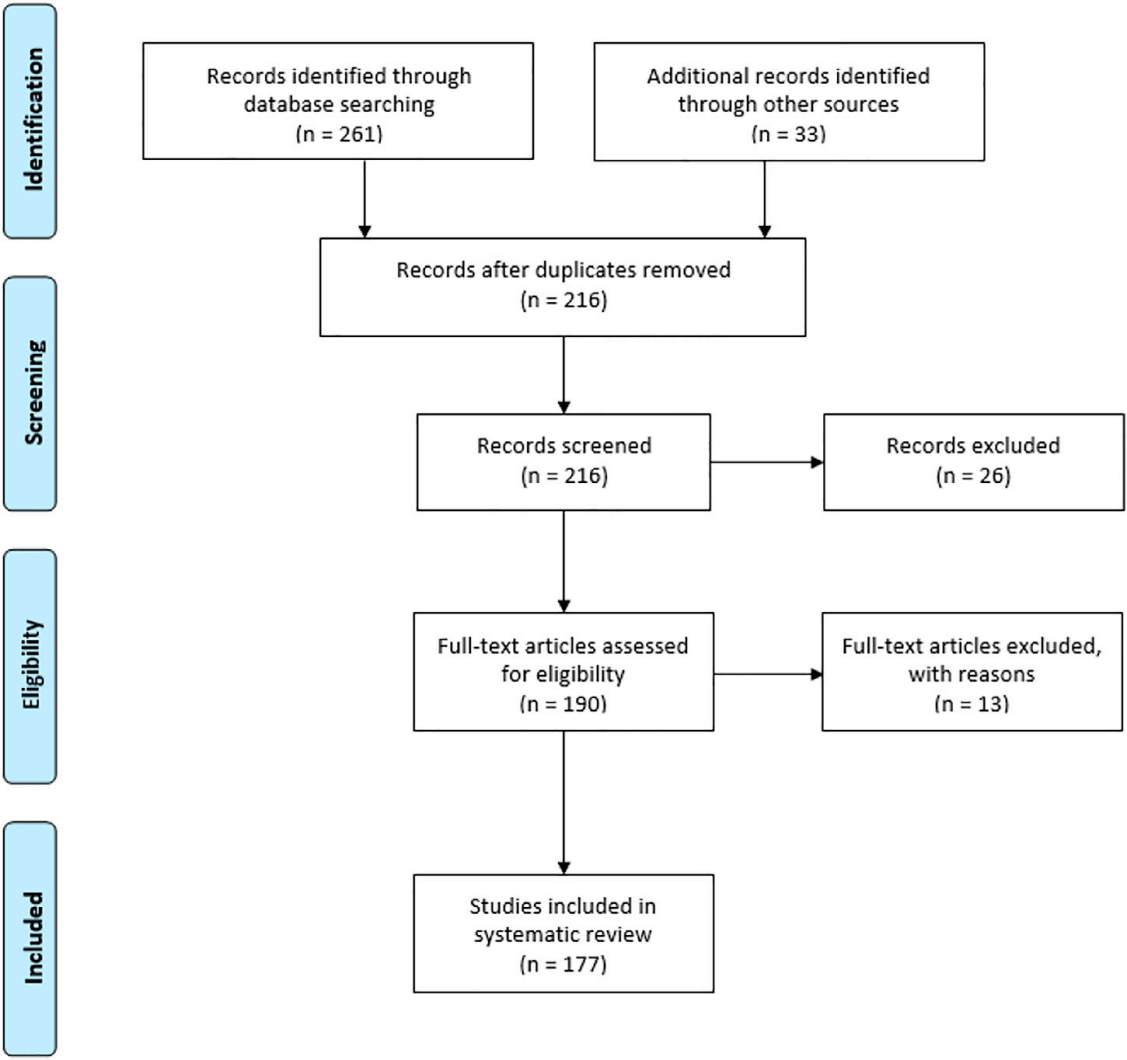
Flow diagram of preferred reporting of items for systematic reviews and meta-analysis.

Similarly, 26 records were excluded from screened records, and 13 full-text articles were excluded due to improper validation from eligible articles. Finally, the authors included 177 articles from peer-reviewed journals and books in the present study. The detailed PRISMA is well represented in Figure 2.

### 2.2 Inclusion and Exclusion Criteria

Information obtained from sources was analyzed carefully based on the below-mentioned inclusion and exclusion criteria.

#### 2.2.1 Inclusion Criteria

1. Full-text studies related to shikonin sources, isolation, biosynthesis, chemical synthesis, pharmacology, patent, and toxicology
2. Pre-clinical and clinical studies related to shikonin
3. Review articles
4. *In vitro* and *in vivo* studies of toxicology of shikonin were included

#### 2.2.2 Exclusion Criteria

1. Abstract only or half manuscript
2. Duplicate literature
3. Lacking complete botanical information of shikonin sources
4. Lacking transparent methodology and objectives were excluded.

### 2.3 Data Extraction and Review Process

After importing the electronic database from sources to Mendeley reference software, articles were analyzed, and duplicate articles were removed. The article was then analyzed and included based on inclusion and exclusion criteria. Analysis of articles revealed that shikonin and its derivatives have other botanical sources apart from well-known *Lithospermum erythrorhizon Siebold & Zucc.* These botanical sources can be found worldwide, but the significant concentration is in Asia, as concluded from the literature available*.* Furthermore, the isolation of shikonin and its derivatives from these botanical sources has also been a study of interest, such as the chromatographic separation method (Azuma et al., 2016), ultrasound-assisted method (Huang et al., 2020), and ultrasound-assisted ionic liquid solid-liquid extraction (Sun et al., 2019). Furthermore, biosynthesis and Genetical synthesis of shikonin has been an area of interest for various research; hence several articles are available proposing possible biosynthetic pathways or catalysts like CYP76B100 or CYP76B101 (Cytochrome P450s catalyst) (Widhalm and Rhodes, 2016), CYP76B74 (Wang et al., 2019), deoxyshikonin hydroxylases (DSH1/DSH2) (Song et al., 2021), and other enzymes. Based on the data available, it is clear that sources of shikonin and extraction to biosynthesis still have several more scopes to work on, which can ultimately allow a better understanding of the pharmacology of shikonin.

The overexploitation of shikonin botanical sources has increased the chemical synthesis of shikonin and its derivatives. Several synthetic pathways have been analyzed from the past till now. The majority number of articles available are based on the pharmacology of shikonin and its derivatives. Several clinical and pre-clinical studies were obtained from ClinicalTrails.gov. These studies reveal the growing importance of shikonin in the field of drug discovery against various diseases. Several patents filed were based on pharmacology and other use of shikonin and its derivatives. Interestingly, fewer articles that present toxicology or the safety of shikonin as a potential drug is available, requiring a further and deeper study of toxicology.

## 3 Natural Sources of Shikonin and Their Isolation

**FIGURE 3:**
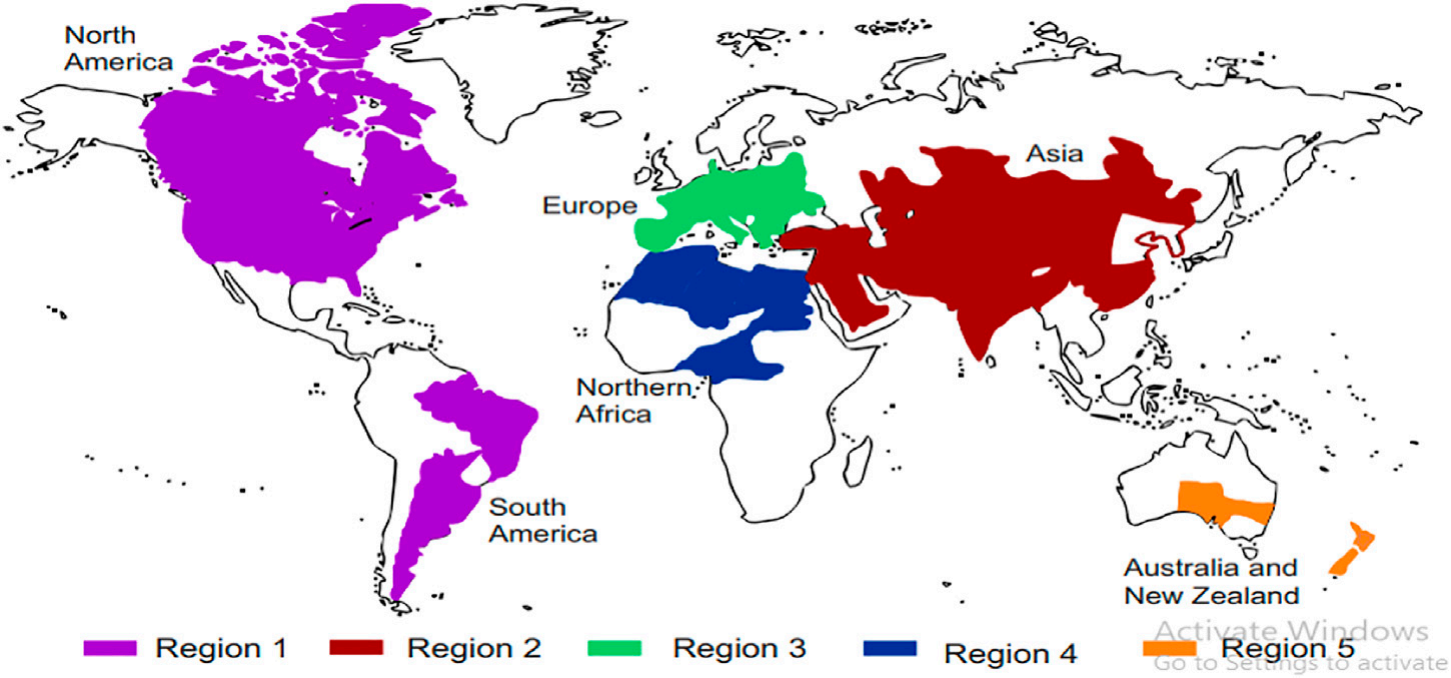
Distribution of shikonin-producing plants around the world. Region 1 — North and South America, Region 2 — Asia, Region 3 — Europe, Region 4 — Africa, and Region 5 — Australia and New Zealand (Table 2).

**FIGURE 4:**
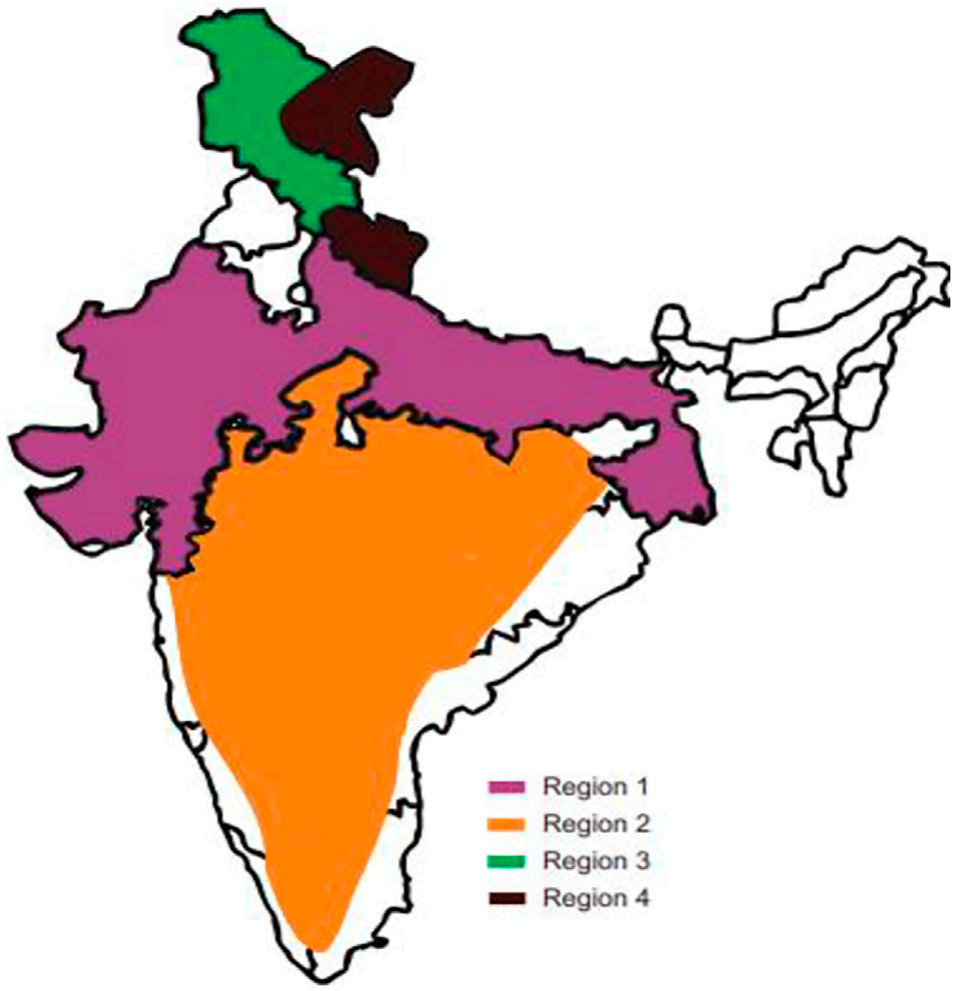
Distribution of shikonin-producing plants across India. Region 1 — Rajasthan, Gujrat, Ganga plains, Region 2 — Peninsular India, Region 3 and 4 — Spiti (cold desert areas of Himachal Pradesh), Uttarakhand, Leh and Ladakh. (Refer to Table 3).

Natural sources of shikonin and its chemical derivatives, their geographical distribution across the globe, and their traditional and modern pharmacological uses are presented in Table 1. Many more plants’ sources of shikonin and its derivatives can be found worldwide. Detailed distribution of natural sources of shikonin around the world and India, along with their nomenclature, are discussed in Table 2 and Table 3, respectively, and well represented in Figure 3 and Figure 4, respectively.

**TABLE 1:** Natural sources of shikonin and its derivatives.

Sr. No.	Botanical names	Geographical distribution	Compounds extracted	Traditional uses	Pharmacological uses
1.	*Lithospermum erythrorhizon Siebold and Zucc*. (Assimopoulou et al., 2009)Common name-Zi Cao and purple gromwell Lu et al. (2011)	Japan, China, and Korea (Naito et al., 1995)	Shikonin, isobutyl shikonin, naphthalenedion, β,β-dimethyl-acryl shikonin, A mixture of two caffeic acids, β-sitosterol Han et al. (2008)	Treatment of burns, skin disease, sore throat, measles, cuts, dyes, food colorant Lu et al. (2011)	Antioxidant, antifungal, antitumor, anti-HIV Han et al. (2008)
2.	*Arnebia Decumbens Ventenat*) *Cosson and Kralik* (Al-Mussawi 2010)	Kuwait deserts (Afzal and Al-Oriqat 1986) and south-eastern part of Algeria (Eddine et al., 2016)	Shikonin acetate, shikonin (Afzal and Al-Oriquat 1986), shikonin isovalerate (Afzal and Al-Oriqat 1986), pyrrolizidine alkaloids (El-Shazly and Wink 2014) flavonoids (Eddine et al., 2016)	-	Antimicrobial, antioxidant (Eddine et al., 2016), and anti-inflammatory (Al-Mussawi 2010)
3.	*Onosma leptantha Heldr.* (Kundakovic et al., 2006)	South Greece (Kundakovic et al., 2006)	Acetylshikonin, iovaleryl shikonin, β,β-dimethylacryl shikonin (Kundakovic et al., 2006)	-	Cytotoxic and anti-inflammatory (Kundakovic et al., 2006)
4.	*Arnebia hispidissima (Lehm.)*	Egypt (El-Sayed 2010), Deserts of Rajasthan (India), Gujrat (India), Ganga plains (India) (Jain et al., 2000)	Arnebin-7,Arnebin-1, tiglic acid, arnebinol, alkannin, cycloarnebin-7, shikonin (Al-Mussawi 2010)	Used in India for treatment of throat, fever, tongue as an ailment and also used as a tonic for the whole body (Jain et al., 2000)	Anti-ulcer, antibacterial, antioxidant. (Phulwaria and Shekhawat 2013)
5.	Singh et al. (2003)				
	*Lithospermum canescens* (Michx.) *Lehm.* (Pietrosiuk and Wiedenfeld (2005) Common name - hoary puccoon (Kittelson et al., 2006)	The southern part of Canada and northern part of the United States Pietrosiuk and Wiedenfeld (2005)	Acetylshikonin, isovaleryl shikonin, isobutyryl shikonin, pyrrolizidine alkaloids, α-methylbutyryl- shikonin Pietrosiuk and Wiedenfeld (2005)	Used as body dye	Anticancer, antibacterial, antifungal, immune-stimulating, and inflammatory. Pietrosiuk and Wiedenfeld (2005)
6.	*Arnebia euchroma* (Royle) Johnst. (Assimopoulou et al., 2009) Common name-Sorkh Giyah or Heveh Choaeh (Ghasemi Pirbalouti et al., 2009)	Indian western Himalaya (Singh et al., 2012) Iran (Ghasemi Pirbalouti et al., 2009)	Monoterpenes, 2.1,4-naphthoquinones, organic acids, pyrrolizidine alkaloids, arnebiabinone, octyl ferulate (Liu et al., 2010), shikonin derivatives (Ghasemi Pirbalouti et al., 2009)	Skin disease and anti-inflammatory effect (Ghasemi Pirbalouti et al., 2009)	Anti-inflammatory, antitumor, and antimicrobial (Ghasemi Pirbalouti et al., 2009)
7.	*Alkanna tinctoria (L.) Tausch* (Assimopoulou et al., 2009)Common name– Ratanjot (Singh et al., 2012)	South Europe (Mita et al., 1994)	Shikonin, alkannin, esters of shikonin and alkannin (Assimopoulou and Papageorgiou 2005)	Romans used it to dye their robes, and for its anti-inflammatory effect (Mita et al., 1994).	Antibacterial, anti-inflammatory, wound healing and antioxidant (Mita et al., 1994)
8.	Dyer’s alkanet				
	*Onosma echioides (L.) L.* (Nikita et al., 2015)	Apennines hills of Italy (Mengoni et al., 2006)	Alkannins and shikonins (Nikita et al., 2015)	**-**	Wound-healing (Nikita et al., 2015)
9.	*Jatropha glandulifera Roxb*Common name-Ratanjot (Ballantine 1969)	Pakistan (Ballantine 1969)	3,3-dimethylacrylylshikonin, acetylshikonin (Ballantine 1969)	Rheumatic pains (Ballantine 1969)	Anti-inflammatory, wound healing, antioxidant activity. (Ballantine 1969)
10.	*Lithospermum officinale L*Common name- Pearl gromwell (Al-Snafi 2019)	Asia and Europe (Al-Snafi 2019)	Shikalkin, shikonin, pyrrolizidine alkaloids, polyphenolic acids (Al-Snafi 2019)	Urogenital diseases, and antidiarrhoeal drugs. (Al-Snafi 2019)	Anticancer, antioxidant, and wound-burning healing. (Al-Snafi 2019)

**TABLE 2:** Sources of shikonin around the world.

S. No.	Region	Continent	Name of plants	References
1	Region-1	North and South America	*Echium plantagineum L.*	Piggin (1978)
			*Lithospermum canescens (Michx.) Lehm.*	Pietrosiuk and Wiedenfeld (2005)
			*Lithospermum officinale L.*	Winterhoff (1993)
2	Region-2	Asia	*Arnebia Decumbens (Ventenat) Cosson&Kralik*	Afzal and Al-Oriqat (1986)
			*Arnebia hispidissima (Lehm.)*	El-Sayed (2010)
			*Arnebia euchroma* (Royle) Johnst	Kumar et al. (2021)
			*Arnebia guttata Bunge*	Song et al. (2019)
			*Alkanna tinctoria (L.) Tausch*	Mita et al. (1994)
			*Echium plantagineum L.*	Piggin (1978)
			*Eritrichium incanum (Turcz.) A. DC.*	Ovchinnikova (2008)
			*Jatropha glandulifera Roxb*	Ballantine (1969)
			*Lithospermum erythrorhizon Siebold and Zucc*	Naito et al. (1995)
			*Lithospermum officinale L.*	Al-Snafi (2019)
			*Onosma caucasica Levin*	Daironas et al. (2014)
			*Onosma bulbotrichum DC*	Mehrabian et al. (2011)
3	Region-3	Europe	*Alkanna tinctoria (L.) Tausch*	Mita et al. (1994)
			Buglossoides arvensis (L.) *I. M. Johnst.*	Olennikov et al. (2020)
			*Cynoglossum officinale L.*	Papageorgiou et al. (1999)
			*Echium plantagineum L.*	Piggin (1978)
			*Lithospermum officinale L.*	Al-Snafi (2019)
			Rindera graeca Boiss. and amp	Kanou et al. (1990)
			*Onosma echioides (L.)*	Mengoni et al. (2006)
			*Onosma leptantha Heldr*	Damianakos et al. (2014)
			*Onosma caucasica Levin*	Daironas et al. (2014)
4	Region-4	Northern Africa	*Arnebia Decumbens (Ventenat) Cosson and Kralik*	Eddine et al. (2016)
			*Arnebia hispidissima (Lehm.)*	El-Sayed (2010)
			*Alkanna tinctoria (L.) Tausch*	Mita et al. (1994)
			*Echium plantagineum L.*	Piggin (1978)
5	Region-5	Australia and New Zealand	*Echium italicum* L.	Seaman and Dellow (1985)
			*Echium plantagineum L.*	

**TABLE 3:** Abundance of shikonin in India.

S. No.	Region	States	Name of plant	References
1	Region-1	Rajasthan, Gujrat, Ganga plains	*Arnebia hispidissima (Lehm.)*	Jain et al. (2000)
2	Region-2	Peninsular India	*Jatropha glandulifera Roxb*	Ballantine (1969)
3	Region-3	Spiti (cold desert areas of Himachal Pradesh), Uttarakhand, Leh and Ladakh.	*Arnebia euchroma (Royle) Johnst Arnebia guttata Bunge*	Kumar et al. (2021)
				Song et al. (2019)
4	Region-4	Spiti (cold desert areas of Himachal Pradesh), Uttarakhand, Leh and Ladakh.	*Arnebia euchroma (Royle) Johnst*	Kumar et al. (2021)

**SCHEME 1:**
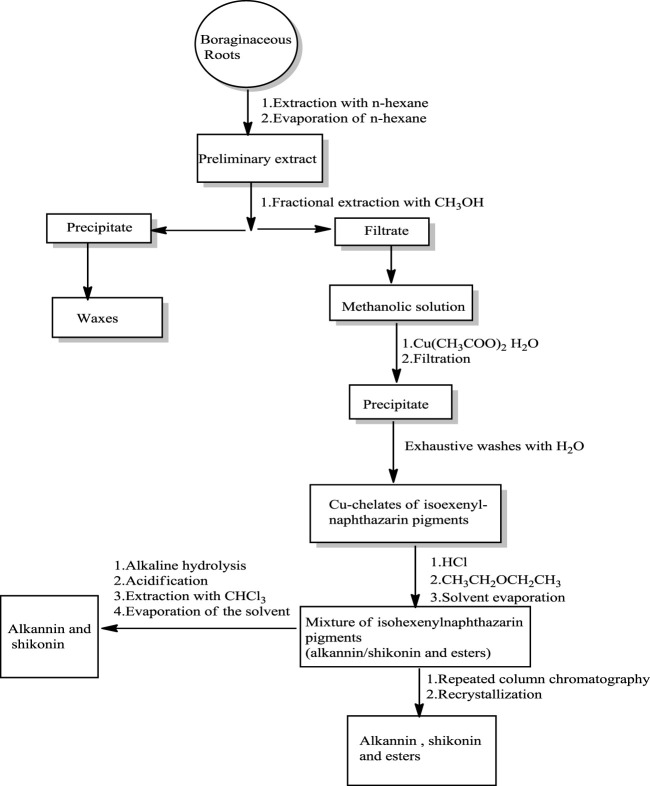
Isolation and purification of shikonin (Assimopoulou et al., 2009).

Two Japanese chemists, Majima and Kuroda (1922) first isolated shikonin in its acetate form from the roots of *L. erythrorhizon* (Papageorgiou et al., 1999). Abdulameer and Al-Mussawi (Al-Mussawi, 2010) reported a method for isolating shikonin from *A. decumbens* (Scheme 1) using *n*-hexane and methanol or petroleum ether. The roots of *A. decumbens* were dried, powdered, and firstly extracted with hexane, and the resulting extract was purified over silica gel eluting with ethyl acetate and petroleum ether (Al-Mussawi, 2010).

A new, improved isolation method of shikonin from the roots of *L. erythrorhizon* was proposed by Azuma et al. (2016). The shikonin was isolated by chromatographic separation using hexane as solvent (Azuma et al., 2016), resulting in a 2% yield from dried powder roots of *L. erythrorhizon* (Azuma et al., 2016). Huang et al. (2020) used an ultrasound-assisted method of extraction of shikonin from *A. euchroma*, mixed with an ethanol solution. The shikonin yield was determined using high-performance liquid chromatography (HPLC) as 1.26% using the following conditions: 39°C; ultrasound power of 93 W; extraction for 87 min; and the ratio of liquid (ethanol): solid (roots) was 11:1 (Huang et al., 2020). Advance study on solid-phase extraction of shikonin from *Alkanna tinctoria* roots using Molecularly Imprinted Polymers (MIPs) targeting *i.e.,* methacrylic acid and 2-diethylaminoethyl methacrylate as functional monomers showing strong affinity toward basic functionality in solution association study between shikonin and acidic-basic functional monomers (Tsermentseli et al., 2013). In polar conditions, the selectivity was minimal, whereas methacrylic acid was still more selective toward shikonin. While in the non-polar solvent 2-diethylaminoethyl methacrylate-based polymer was more selective toward shikonin. Overall, the recovery came up to 72% in the hexane extract of *Alkanna tinctoria* roots (Tsermentseli et al., 2013). Sun et al. (2019) used ultrasound-assisted ionic liquid solid-liquid extraction and an aqueous two-phase extraction method to extract shikonin and its derivatives from *A. euchroma.* The shikonin was extracted in a 1-butyl-3-methylimidazolium tetrafluoroborate solvent. Shikonin and derivatives went into the upper layer. Analysis by HPLC showed a high yield of 90%–97% shikonin (Sun et al., 2019). Thus, it appears to be the best and most efficient extraction method to date.

## 4 Biosynthetic Pathways of Shikonin

**SCHEME 2:**
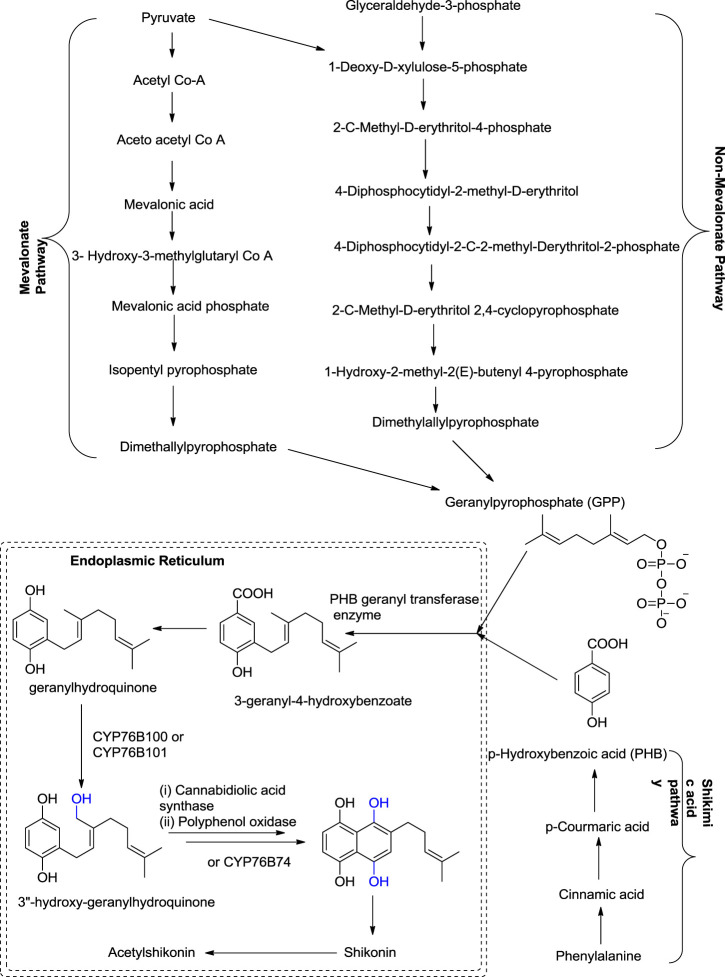
Biosynthesis of shikonin (Widhalm and Rhodes, 2016; Gaisser and Heide, 1996; Song et al., 2020; Wang et al., 2019b; Takanashi et al., 2019; Song et al., 2021).

Shikonin and its derivatives are biosynthesized from two precursors: p-hydroxybenzoic acid (PHB) and geranyl pyrophosphate (GPP) (Boehm et al., 2000) in the endoplasmic reticulum (Muhenweg et al., 1998) of the cell of the plant *L. erythrorhozion* (Scheme 2). PHB and GPP are produced from phenylalanine and acetyl CoA, respectively. Acetyl CoA combines with HMG (3-hydroxy-3-methylglutaryl), giving 3-hydroxy-3-methylglutaryl-coenzyme A (HMG-CoA), which is catalyzed by an HMGR (HMG reductase) enzyme-producing mevalonate, which further produces GPP (Muhenweg et al., 1998; Lange et al., 1998). The GPP is synthesized either by mevalonate or non-mevalonate pathways (Kumar et al., 2014; Widhalm and Rhodes, 2016), while PHB is produced by the shikimic acid pathway (Kumar et al., 2016). GPP and PHB, in the presence of PHB geranyl transferase enzyme, generate the first intermediate of shikonin biosynthesis, i.e., 3-geranyl-4-hydroxybenzoate (GBA) (Lange et al., 1998; Widhalm and Rhodes, 2016), subsequently producing geranylhydroquinone finally leading to the synthesis of shikonin, which moves out of the endoplasmic reticulum. Geranylhydroquinone in the presence of CYP76B100 or CYP76B101 (Cytochrome P450s catalyst) produces 3″-hydroxy-geranylhydroquinone, which can generate dihydroechofuran as a minor product along with the production of shikonin (in the presence of CYP76B74 catalyst) (Widhalm and Rhodes, 2016; Scheme 2).

**SCHEME 3:**
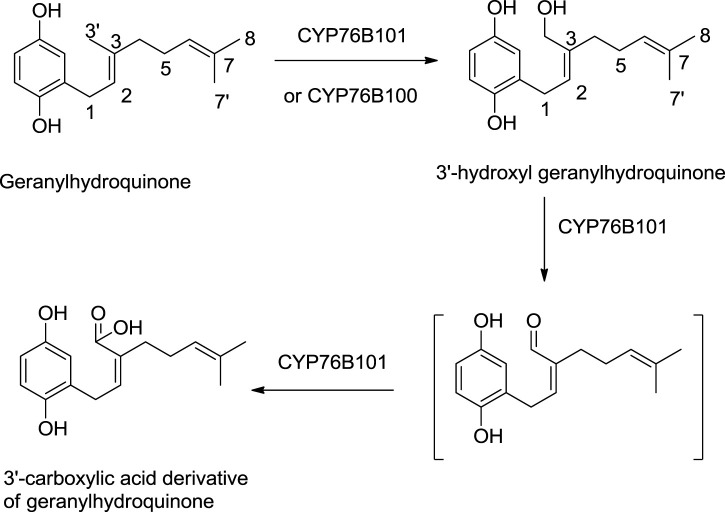
CYP76B100 and CYP76B100 catalyst role in shikonin biosynthesis (Song et al., 2020).

A study by Song et al. (2020) shows the significant role of cytochrome P450s catalysts, i.e., CYP76B100 and CYP76B101, in the biosynthesis of shikonin. First, the geranyl hydroquinone side chain is hydroxylated at the C-3″ position by CYP76B100, forming 3′-hydroxy-geranylhydroquinone, which undergoes oxidation at the C-3″ position with CYP76B101 catalyst producing a 3″- carboxylic acid derivative of geranylhydroquinone and 3″-hydroxy-geranylhydroquinone, leading to shikonin and its derivatives (Scheme 3; Song et al., 2020).

Wang et al. (2019) reported that CYP76B74 catalyzes the important hydroxylation step of shikonin biosynthesis, i.e., conversion of 3″-hydroxy-geranylhydroquinone into shikonin. CYP76B74 is a cytochrome P450 catalyst belonging to the CYP76B subfamily. The activity of CYP76B74 in biosynthesis effectively produces shikonin and further facilitates ring closure to produce dihydroechinofurans (Wang et al., 2019).

**SCHEME 4:**
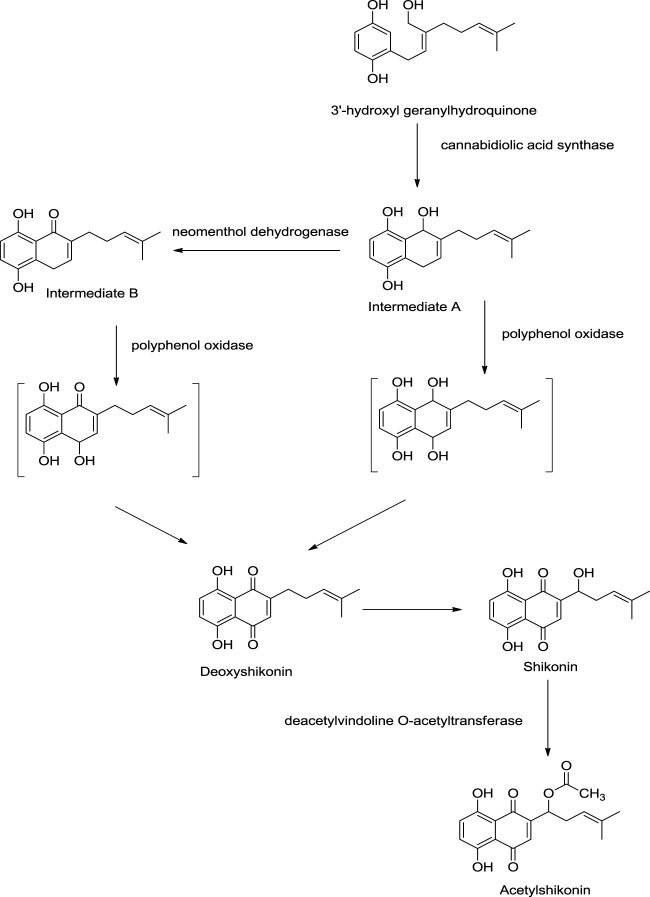
Role of polyphenol oxidase, neomenthol dehydrogenase like proteins, and cannabidiolic acid synthase in shikonin biosynthesis (Takanashi et al., 2019).

Recent studies have shed light on new enzymes involved in shikonin biosyntheses like polyphenol oxidase, neomenthol dehydrogenase-like proteins, and cannabidiolic acid synthase (Scheme 4). 3″-Hydroxyl geranylhydroquinone is converted into intermediate A *via* cyclization using cannabidiolic acid synthase. Further, intermediate A can be converted into intermediate B by neomenthol dehydrogenase catalyst. Both intermediate A and intermediate B undergo oxidation using polyphenol oxidase synthesizing deoxyshikonin, which further yields shikonin. Shikonin converts into acetylshikonin using deacetylvindoline O-acetyltransferase (Takanashi et al., 2019).

**SCHEME 5:**
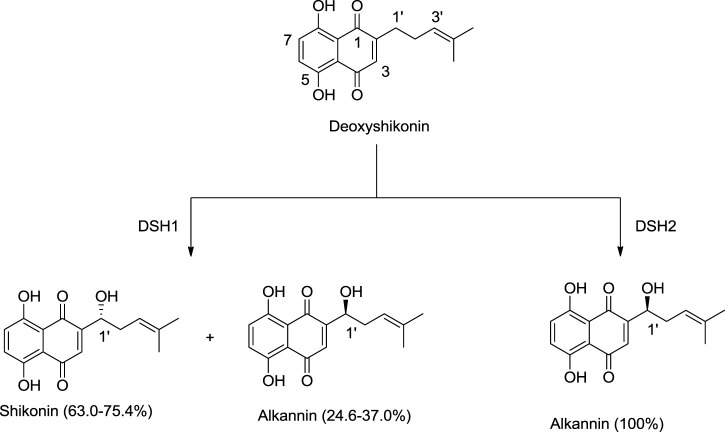
Deoxyshikonin hydroxylases (DSHs) catalyzing deoxyshikonin to synthesize shikonin and alkannin (Song et al., 2021).

Song et al. (2021) discovered new cytochrome P450 monooxygenases, namely deoxyshikonin hydroxylases (DSH1/DSH2), belonging to the CYP82AR subfamily (Song et al., 2021). DSH1 and DSH2 catalyze deoxyshikonin to convert into shikonin. DSH1 hydroxylates deoxyshikonin into shikonin, while DSH2 converts deoxyshikonin into alkannin (Scheme 5; Song et al., 2021).

Further studies have shown that genes like LeMYB1 in *L. erythrorhozion* control the synthesis of the shikonin biosynthetic pathway. The gene expression results in the encoding of enzymes like phenylalanine ammonialyase, HMGR, p-hydroxybenzoate-geranyltranferase, and regulating factors like *L. erythrorhozion* dark-inducible gene (LeDI-2) and *L. erythrorhozion* pigment callus-specific gene (LePS-2), which take part in the biosynthesis of shikonin (Zhao H. et al., 2015). This study further indicates the possibility of overexpression of LeMYB1 *via* genetic engineering, which can, in turn, increase the production of shikonin in *L. erythrorhozion* (Zhao H. et al., 2015).

Oshikiri et al. published a study in 2020 showing the potential of BAHD acyltransferases on shikonin biosynthesis from *L. erythrorhozion*. The two BAHD acyltransferases- LeSAT1 (shikonin O-acyltransferase) and LeAAT1 (alkannin O-acyltransferase) enzymes show high acylation capability and are specifically producing shikonin and alkannin derivatives, respectively (Oshikiri et al., 2020).

### 4.1 Genetic Engineering on Shikonin Biosynthesis

**SCHEME 6:**
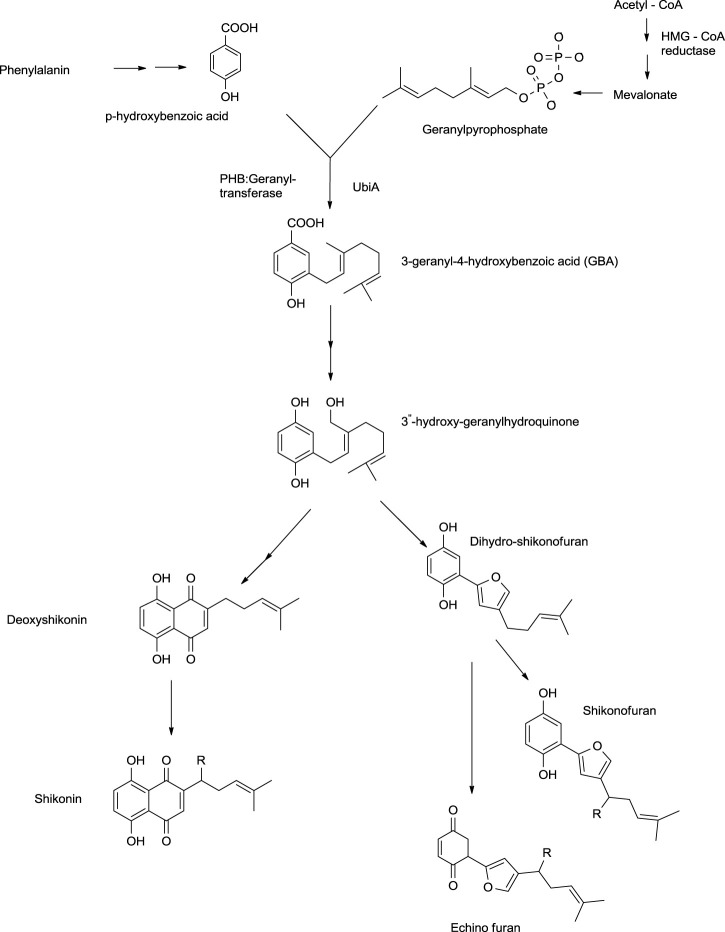
Genetically engineered biosynthesis of shikonin.

The shikonin biosynthetic pathway was altered (Boehm et al., 2000) by introducing the ubiA gene of *E. coli,* which codes for 4-hydroxybenzoate-3-polyprenyltransferase, a membrane-bound enzyme catalyzing the emergence of GBA using GPP substrate. For targeting the resulting peptide, the ubiA gene was mixed with two sequences to the endoplasmic reticulum (ER) and introduced in *L. erythrorhizon* through *Agrobacterium rhizogenes.* As a result, the production of shikonin increased by 22% as the enzymatic activity of the pathway increased (Scheme 6).

A study performed by Pietrosiuk (Pietrosiuk et al., 2006) indicated that when strains of *A. rhizogenes* were introduced into the root culture of *L. canescens* a significant increase in shikonin derivatives’ production was observed. The *A. rhizogenes* ATCC 15834 strain increases the yield of acetylshikonin and isobutrylshikonin and hence increased the biomass of hairy root culture up to *ca* 10% of *L. canescens* (Pietrosiuk et al., 2006)*.* Tatsumi et al. (2020) developed an effective method for transforming hairy roots of *L. erythrorhizon* using a *Rhizobium rhizogenes* strain A13 (Tatsumi et al., 2020). The *R*. *rhizogenes* strain A13 was infected in the leaf and stem of *L. erythrorhizon*, which encodes for a green fluorescent protein. This transgene expression was monitored, and transformation efficiency was found to be 50%–70% higher than in previously reported studies (Tatsumi et al., 2020). This method will help in a better molecular-level understanding of the biosynthesis of shikonin and its derivatives in *L. erythrorhizon* (Tatsumi et al., 2020).

Another study demonstrated that the ubiC bacterial gene, when introduced in *L. erythrorhizon,* converts chorismate to 4-hydroxy-benzoate via encoded chorismate pyruvate-lyase enzyme. The process was achieved under the control of (octopine synthase)_3_ mannopine synthase promoters. Thereby reducing the steps in the usual biosynthetic pathway. The activity of HMGR was also analyzed by introducing HMGR1 of *Arabidopsis thaliana* in *L. erythrorhizon* under similar conditions, as mentioned above. Despite the overexpression of genes, there was no significant change in shikonin production. This result can be due to the downregulation or simultaneous overexpression of genes altering the biosynthesis pathway of shikonin (Köhle et al., 2002).

A recent study reported a higher yield of acetylshikonin in *E. plantagineum* by overexpression of cloned EpGHQH1 (geranylhydroquinone 3″-hydroxylase candidate gene) (Fu et al., 2021). Geranylhydroquinone 3″-hydroxylase enzyme was found to convert geranylhydroquinone into 3″- hydroxyl geranylhydroquinone, an essential step in shikonin biosynthesis in *E. plantagineum* (Fu et al., 2021). EpGHQH1 overexpression increased acetylshikonin production by 2.1 fold higher than average accumulation in *E. plantagineum* (Fu et al., 2021). Further, it was reported that the introduction of bacteria (specifically *Chitinophaga* sp.*, Allorhizobium* sp.*, Duganella* sp.*, and Micromonospora* sp.) in the hairy root culture of *A. tinctoria* visibly increased the production of shikonin/alkannin (Rat et al., 2021).

### 4.2 Shikonin Production From Cell Culture of Plants Sources

*L. erythrorhizon* roots’ outer surface (bark) contains purple-red coloured compounds, i.e., shikonin and its derivatives (Fujita, 1988). Yazaki et al. (1987) worked on callus cultures of *L. erythrorhizon* and successfully produced shikonin and its derivatives. Later, Fujita (Fujita 1988) worked on suspension and callus culture in Linsmaier and Skoog medium (LS). Callus culture is an unorganized mass of cells produced in an artificial nutrient medium from plant cells. In contrast, suspension culture is the mass of cells suspended and grown in an agitated liquid medium. Suspension culture was not found to produce shikonin, whereas, in callus culture, shikonin derivatives were produced (Hara et al., 1987). The reason was found to be the supply of oxygen in callus culture accompanied by regular nutrient supply without agar, also called M9 medium (minimal growth medium used for bacterial cultures) (Hara et al., 1987). As the oxygen concentration increased in callus culture, cell growth enhanced, producing shikonin (Hara et al., 1987). While the suspension culture was found to contain ammonium ions produced by glutamine in its medium, this effect represses shikonin synthesis **(** Table 4) (Yazaki et al., 1987).

**TABLE 4:** Shikonin production in the presence of ammonium ions in a different medium (Yazaki et al., 1987).

Sr. No.	Medium	NH_4_^+^ (μmol/g)	Growth increase of cell (grams of fresh weight or g fr. wt.)	Shikonin produced
1.	LS	4.75	5.1	0
2.	LS + Cu^2+^	9.49	5.5	0
3.	LS + Cu^2+^- NH_4_^+^ + NO_3_^−^	0.45	6.9	11.8
4.	LS (agar medium)	11.93	5.5	28.8
5.	M9	0.33	2.1	149.5

### 4.3 Other Methods for the Manipulation of Shikonin Production

Irradiation of suspension culture of *L. erythrorhizon* with low to high doses of gamma radiation provided an increase in the yield of shikonin (Chung et al., 2006). *p*-Hydroxybenzoic acid (PHB) geranyltransferase, a significant enzyme in shikonin biosynthesis, stimulated gamma rays, thereby increasing the amount of shikonin produced (Chung et al., 2006). When low energy ultrasound radiation was used on suspended cells of *L. erythrorhizon* shikonin production increased by 60–70%, and shikonin extraction from cells also increased (Lin and Wu, 2002). Hence, the total shikonin yield was increased two to three-fold (Lin and Wu, 2002).

While glucose and fructose have not been found effective in producing shikonin derivatives biosynthesis, a high concentration of sucrose in the cell culture of *L. erythrorhizon* increased the yield of shikonin derivatives (Mizukami et al., 1977). When the cell culture of the plant source of shikonin was treated with ascorbic acid at different doses, it was found that 10–4 M ascorbic acid-dosed cell culture produced maximum shikonin (1.08 mg/g) (Mizukami et al., 1977). Treatment of cell culture of the plant source with L-phenylalanine at different doses increased shikonin production (Mizukami et al., 1977). When a cell suspension of *Arnebia* spp. was observed under dark and light, it was found that the maximum content of shikonin was obtained in dark conditions. In contrast, in the light conditions, the percentage yield of shikonin was less (Gupta et al., 2014). Hence, light inhibits shikonin production (Gupta et al., 2014).

Another study performed by Malik et al. (2011) on *A. euchroma* demonstrated the physio-chemical effect on shikonin derivatives production in cell suspension culture of the leaf of *A. euchroma* (Malik et al., 2011)*.* Cell suspension culture was conducted under *in vitro* conditions using MS medium along with 10.0 mM of 6-benzylaminopurine (BAP) and 5.0 mM of indole-3-butyric acid (IBA) (Malik et al., 2011). The physiochemical factors taken into consideration to study its effect on shikonin derivatives production in cell suspension were temperature, light, sucrose, and pH (Malik et al., 2011)*.*

#### 4.3.1 Effect of Temperature on Shikonin Derivative Production

The cell suspension was kept on incubator shaker sets at 20°, 25°, and 30°C to analyze the temperature effect (Malik et al., 2011). The results indicated the maximum production of shikonin derivatives at 25°C after 12 days (586.17 mg/g) (Malik et al., 2011). Shikonin derivative production was 50.80 mg/g FW (fresh weight) at 30°C, while at 20°C, the production was 429.15 mg/g after 14 days incubation period (Malik et al., 2011). Shikonin derivative production was highest at 25°C (Malik et al., 2011). Temperature affects the production of the shikonin derivatives due to changes in the photosynthesis of the plant and the carbon balance (Sonoike, 1998; Bryant et al., 1983). Under adverse conditions, the plant produces secondary metabolites as a defence mechanism. Since these plants produce shikonin derivatives under low temperatures, therefore at 30°C, the production of shikonin derivatives was less (Malik et al., 2011).

#### 4.3.2 Effect of Light on Shikonin Derivative Production

The cell suspension of the leaf of the *A. euchroma* was incubated unde*r* continuous light and complete darkness (Malik et al., 2011). The shikonin derivatives’ content increases under the light condition for the first 4 days, and then there is a decrease in the yield of the shikonin after 4 days, and the lowest yield is on day 12 (1.0 mg/g FW) (Malik et al., 2011). In the dark, the shikonin production was increased to 2.5 times on the fourth and sixth day and reached a maximum on day 12 (542.19 mg/g FW) (Malik et al., 2011). Hence, it was concluded that shikonin production is hindered in the presence of light (Malik et al., 2011). The dark red pigment was found in the cell suspension under dark conditions, while no pigment in the cell suspension under light conditions was seen. This is due to the accumulation of PHB acid and O-glucoside in the cell, which receives light (Malik et al., 2011). The light inactivates the enzyme (flavoprotein), which is required to produce shikonin. The light inhibits the PHB-geranyl transferase activity hence accumulating shikonin precursor, PHB, as its O- glucoside, reducing shikonin derivative production (Malik et al., 2011). Yazaki et al. (2001) experiment isolated *L. erythrorhizon* dark-inducible genes (LeDIs), which are involved in shikonin biosynthesis, hence promoting the synthesis of shikonin in the dark (Yazaki et al., 2001). Similar experiments also give the same result that shikonin production is high at lower rather than higher temperatures due to the expression of LeDI-2 transcription factor (Fang et al., 2016).

#### 4.3.3 Effect of Sucrose on Shikonin Derivatives Production

shikonin derivative production at different concentrations of sucrose (3%, 6%, 9%, and 12% w/v) versus one culture without sucrose (Malik et al., 2011). There was an increase in shikonin production in all the concentrations of the sucrose medium (Malik et al., 2011). Maximum yield (656.14 mg/g FW) was found at 6% sucrose concentration followed by 3% (561.30 mg/g FW) and 9% (176.10 mg/g FW) after a 12 days time span. While at 0% and 12% concentration, the yield was found to be 10.80 and 47.00 mg/g FW, respectively (Malik et al., 2011). A study on *L. erythrorhizon* also demonstrated that high sucrose concentration and L-phenylalanine concentration increased the formation of shikonin derivatives. However, this trend was not seen in the case of glucose and fructose (Mizukami et al., 1977). Another study performed by Hwang et al. (2002) on *L. erythrorhozion* showed that 4% sucrose is optimal for shikonin production in a B5 basal medium (Hwang et al., 2002). The further increase in sucrose concentration increased shikonin production. Hence, for producing the secondary metabolites and shikonin derivatives, sucrose is a good source of carbon and energy.

#### 4.3.4 Effect of pH on the Shikonin Derivative p; Production

Cells were grown in different pH media ranging from pH 5.00–9.50 by adjusting pH with 0.1 M HCl or 0.1 M KOH (Malik et al., 2011). At pH 8.75, maximum shikonin derivatives’ yield was observed (Malik et al., 2011). With increased pH, cell growth decreases, while shikonin production increases (Malik et al., 2011). The pH of the soil sample from where the plant sample was collected was measured at 8.0 and 8.5, and indeed, the maximum yield of the shikonin derivative was found at a pH of 8.75 (Malik et al., 2011). At alkaline pH, p- hydroxybenzoate geranyl transferase activity increased, which is an essential enzyme for shikonin production, increasing shikonin production. The optimum pH for shikonin derivative production ranges from 7.1 to 9.3 (Malik et al., 2011). However, other studies show some contrasting results. Hwang’s study showed that pH does not significantly affect shikonin production; however, pH promotes colour change in the cell culture of *L. erythrorhozion* (Hwang et al., 2002). In the presence of acidic pH, red colour was observed. Malik et al. (2008) discuss the effect of pH on the synthesis of acetyl shikonin in *A. euchroma* (Malik et al., 2008). Their study shows that the pH ranges from 5.0–6.50 (acidic) promote cell growth, and the pH range from 7.25- to 9.50 (alkaline) promoted pigment production in the culture. Moreover, the maximum content of acetylshikonin production was found to be at pH 9.5 (alkaline) (Malik et al., 2008). Hence, pH was found to impact the growth, biosynthesis of shikonin and promote pigmentation (Malik et al., 2008). This change in color depending upon pH changes has helped in developing a pH sensitive indicator for checking the freshness of food like meat and pork (Ezati et al., 2021b).

Overall, it can be concluded that genetically engineered methods of biosynthesis of shikonin yield maximum amounts of shikonin and its derivatives. At the same time, other manipulations can increase shikonin yield compared to the enzymatic method but not to a great extent.

## 5 Chemical Synthesis of Shikonin

### 5.1 Total Synthesis

#### 5.1.1 Shikalkin Synthesis

**SCHEME 7:**
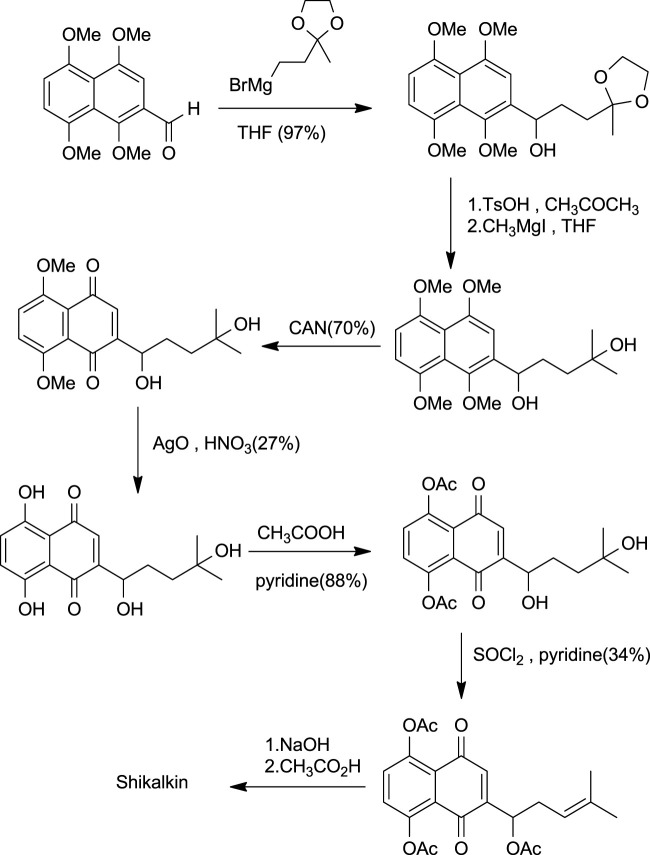
Shikalkin synthesis (Papageorgiou et al., 1999).

Shikalkin (Figure 1), a racemic mixture of alkannin and shikonin, was termed by Brockmann (Terada et al., 1983). Terada gave the first total synthesis of shikonin in the form of shikalkin (Scheme 7). The aldehyde was processed with the Grignard reagent of bromobutan-3-one ethyl acetate, which gives the corresponding acetal. This acetal upon acid hydrolysis easily gets converted into the corresponding ketone, producing a diol in the presence of grignard reagent and methyl iodide. Further, the diol was oxidized using CAN (ammonium cerium (IV) nitrate), giving 1–4 naphthoquinone. Demethylation of naphthoquinone with silver oxide-nitric acid gave tetraol (intermediate). Acetylation of tetraol in pyridine with acetic anhydride produces the triacetate. The triacetate tertiary alcohol group was free for further reactions.

**SCHEME 8:**
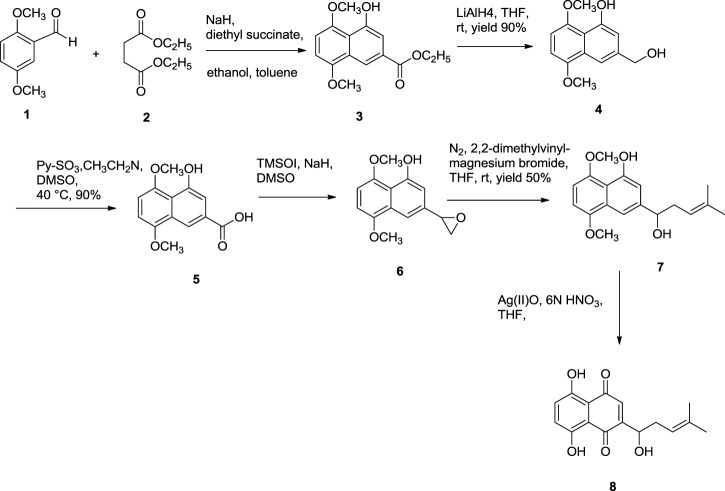
Total synthesis of dl-shikonin. 3: 1,4-dimethoxy-5-hydroxy naphthalene skeleton, 4: naphthol skeleton, 5: aldehyde, 6: 1,4-bismethoxy-5-hydroxynaphthalene epoxides, 7: naphthol skeleton, 8: dl-shikonin (Lu et al., 2008).

**SCHEME 9:**
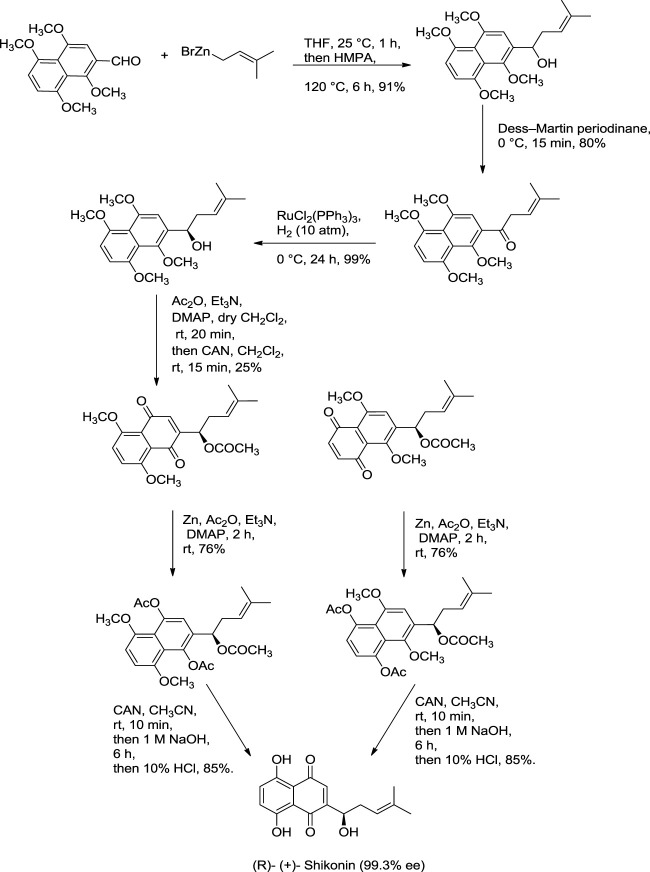
Total synthesis of shikonin (Wang et al., 2012a).

Further dehydration of triacetate’s hydroxyl group in pyridine by thionyl chloride at 38°C for 7 min produces triacetylshikalkin and vinylidene isomer (3:1 ratio). This mixture was separated by silica gel chromatography. Crude crystals of shikalkin were obtained by saponifying triacetylshikalkinin with 1 M NaOH and acidification with acetic acid. A pure sample (+)-shikonin was obtained from repeated purifications and recrystallization processes (Terada et al., 1983). Lu et al. (2008) developed another method for the total synthesis of shikalkin *via* 1,4-bismethoxy-5-hydroxynaphthalene epoxides intermediate formation (Scheme 8). The reaction starts by condensing reactants generating the 1,4-dimethoxy-5-hydroxy naphthalene skeleton, which is subjected to reduction using lithium aluminium hydride (LiAlH_4_) to produce naphthol. Naphthol, when oxidized, gives the aldehyde using pyridine-SO_3_. The sulfur yield methodology was used to produce the 1,4-bismethoxy-5-hydroxynaphthalene intermediate from the aldehyde. Then the Grignard reagent was used to open the epoxide ring of the intermediate yielding naphthol, which undergoes oxidation with AgO (II) in the presence of tetrahydrofuran (THF), producing a moderate yield of dl-shikonin (Lu et al., 2008). Wang et al. also devised a novel total synthetic route utilizing Ru (II) catalyst and finally removing methyl protecting groups, yielding shikonin (47%) in six steps. This synthesis yields 99.3% ee enantiomeric excess of shikonin (Scheme 9) (Wang et al., 2012a).

#### 5.1.2 Torri and Co-Workers Approach

**SCHEME 10:**
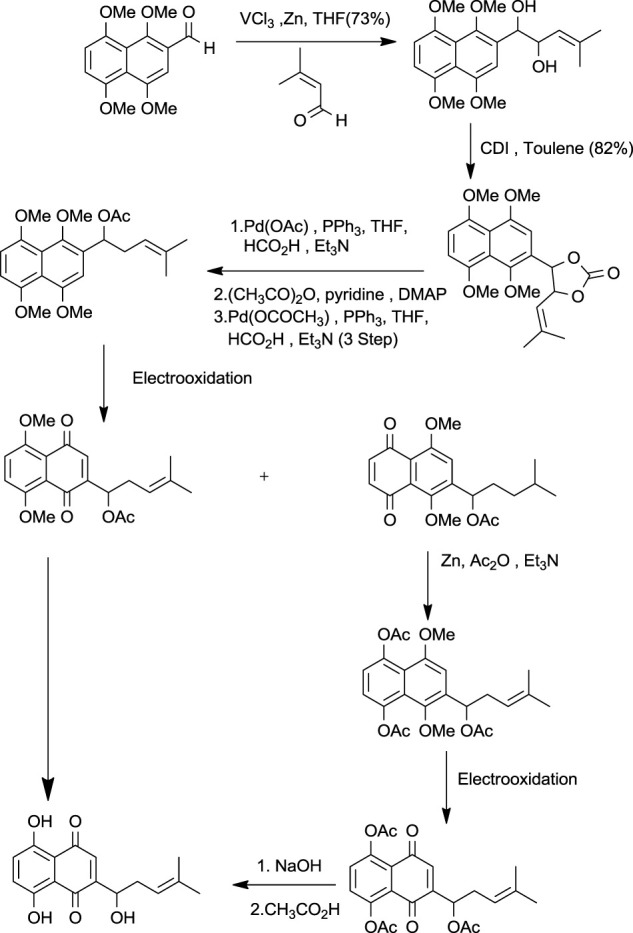
Total synthesis of shikonin by Torri and co-workers (Torii et al., 1995).

Torri and co-workers have reported the synthesis of shikonin (Scheme 10) from formyl derivatives (Papageorgiou et al., 1999). The coupling of 1,4,5,8-tetra methoxy naphthalene-2-carbaldehyde and 3-methyl-2-butenal assisted by vanadium (III) was the initial step for the induction of the side chain of shikonin. Further, by pinacol coupling, 2-(1-hydroxy-4-methyl-3-pentenyl)-1,4,5,8-tetra methoxy naphthalene was formed, and similarly, the carbon-oxygen bond undergoes hydrogenolysis by palladium-catalysis of the diol carbonate at the allylic position. 2-Substituted 1,4,5,8-tetramethoxynaphthalene was electrochemically oxidized, then reduced with zinc giving 5,8-diacetoxy-1,4-dimethoxynaphthalene, which had undergone electrooxidation producing 5,8-diacetoxy-1,4-naphthoquinone, which after alkaline hydrolysis produced dl-shikonin (Torii et al., 1995).

#### 5.1.3 Nicolaou Total Synthesis

**SCHEME 11:**
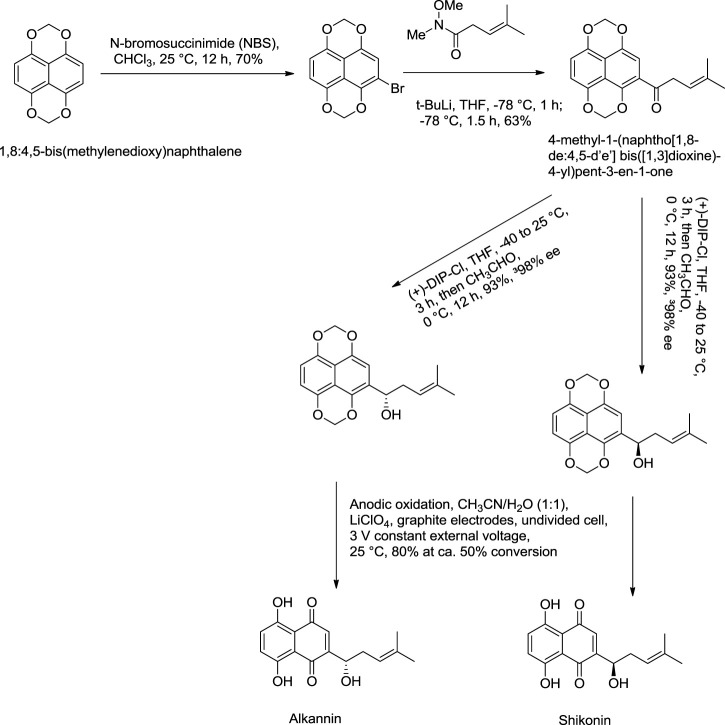
Nicolaou total synthesis of shikonin and alkannin (Nicolaou and Hepworth, 1998).

**SCHEME 12:**
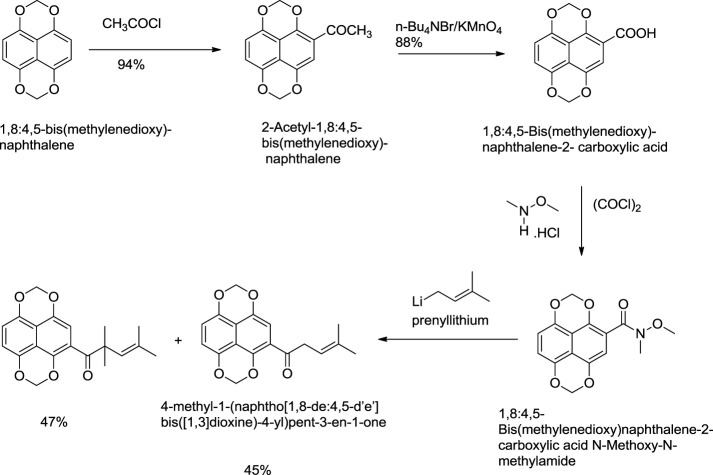
A new synthesis for the preparation of key intermediate for Nicolaou total synthesis (Zheng et al., 2021).

Nicolaou and Hepworth (1998) reported an effective total synthesis of shikonin from 1,8:4,5-bis(methylenedioxy)naphthalene as the starting material and 4-methyl-1-(naphtho [1,8-de:4,5-d’e’] bis([1,3]dioxine)-4-yl)pent-3-en-1-one as an intermediate (Scheme 11). Nicolaou used N-methoxy-N,4-dimethylpent-3-enamide to prepare intermediate in this synthesis route (Nicolaou and Hepworth, 1998). N-methoxy-N,4-dimethylpent-3-enamide was found to be toxic; therefore, a recent study by Zheng et al. (2021) reported a new synthesis for the preparation of key intermediate from the reaction of 1,8:4,5-bis(methylenedioxy)naphthalene-2-carboxylic acid N-methoxy-N-methylamide and prenyllithium (Scheme 12) (Zheng et al., 2021).

### 5.2. Synthesis Through Cycloshikonin

#### 5.2.1 Terada Approach

**SCHEME 13:**
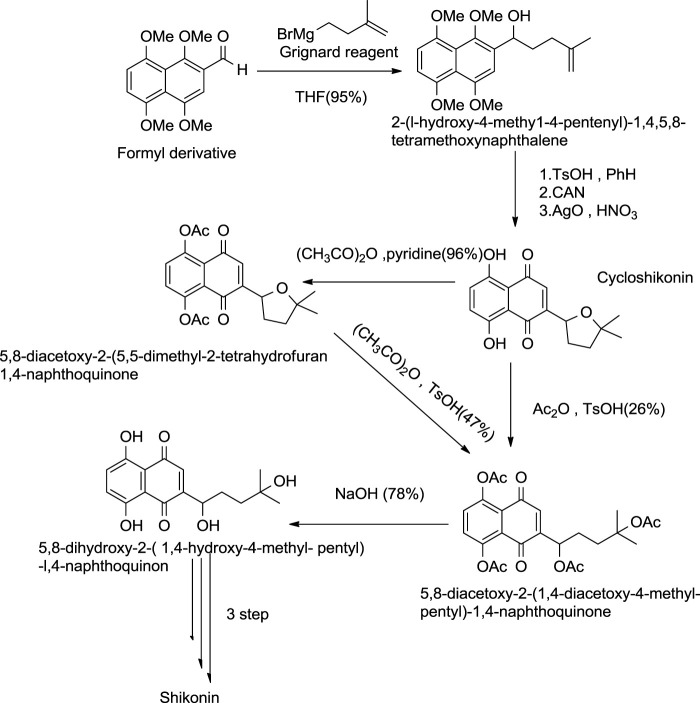
Terada approach by cycloshikonin for the synthesis of shikonin (Tanoue et al., 1987).

This method of formation of shikonin by cycloshikonin was given by Terada and co-workers (Scheme 13) (Tanoue et al., 1987). In this method, intermolecular cyclization of 2-(1-hydroxy-4-methyl-4-pentenyl)-1,4,5,8-tetramethoxynaphthalene occurred, giving cycloshikonin with CAN. Then, Cycloshikonin ring-opening occurs in acetic anhydride with *p*-toluenesulfonic acid-producing 5,8-diacetoxy-24 (1,4-diacetoxy-4-methylpentyl)-1,4-naphthoquinone, which was hydrolyzed by alkali producing shikonin (Tanoue et al., 1987).

#### 5.2.2 Kanematsu Approach

**SCHEME 14:**
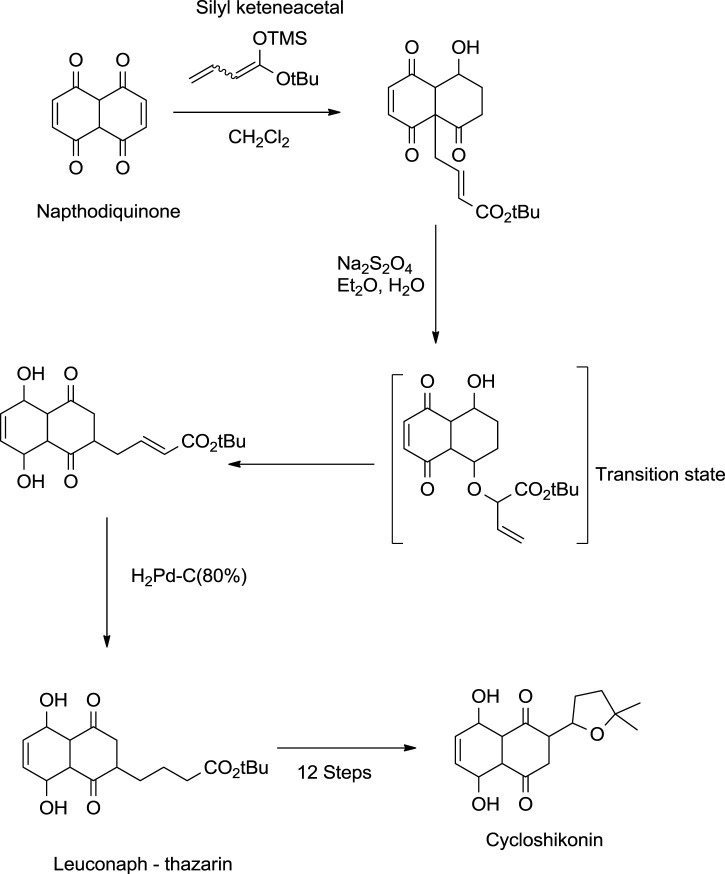
Kanematsu synthesis of cycloshikonin (Aso and Kanematsu, 1993).

Kanematsu also provided a method for synthesising cycloshikonin from naphthoquinone (Scheme 14). In the initial step, naphthoquinone was treated with silyl keteneacetal, which formed a transition complex, which, when reduced with Pd (palladium), gave leuconaphthazarin, finally forming cycloshikonin (Aso and Kanematsu, 1993).

**SCHEME 15:**
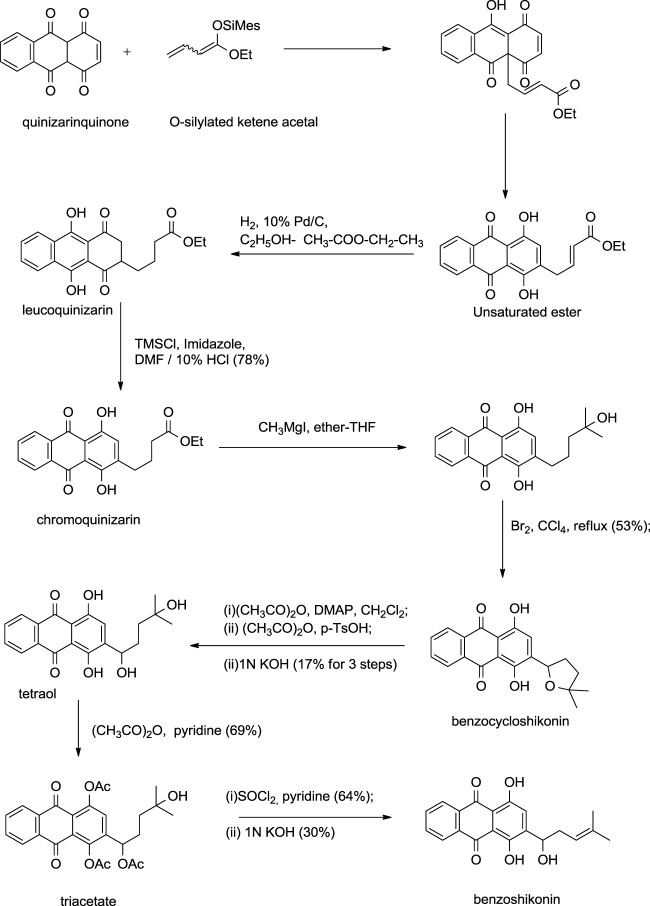
Kanematsu synthesis of benzoshikonin and benzocycloshikonin (Aso et al., 1988).

Kanematsu also developed a similar synthetic route for benzoshikonin and benzocycloshikonin (Aso et al., 1988). The benzo-analog of naphthoquinone was used instead of naphthoquinone as a starting material (Scheme 15) (Aso et al., 1988).

### 5.3. Asymmetrical Approach

#### 5.3.1 Dotz Annulation Approach

**SCHEME 16:**
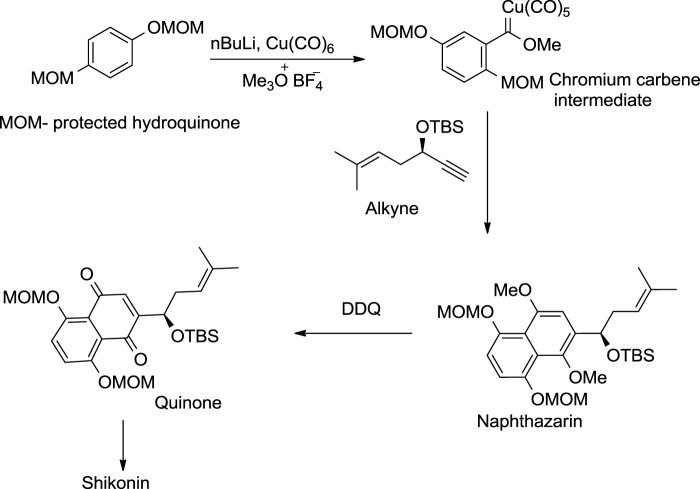
Dotz annulation asymmetrical method for shikonin synthesis (Papageorgiou et al., 1999).

The Dotz annulation reaction was the first asymmetric approach for shikonin synthesis (Scheme 16). The methoxymethyl ether (MOM) protected hydroquinone was first converted into a chromium carbene intermediate, which produced the protected naphthazarin upon treatment with an enantiomeric pure alkyne, whereby the quinone as a phenolic group of naphthazarin underwent selective oxidation. The resulting quinone further provided shikonin through acid hydrolysis (Pulley and Czakó, 2004).

#### 5.3.2 Baurer and Braun Asymmetrical Approach

**SCHEME 17:**
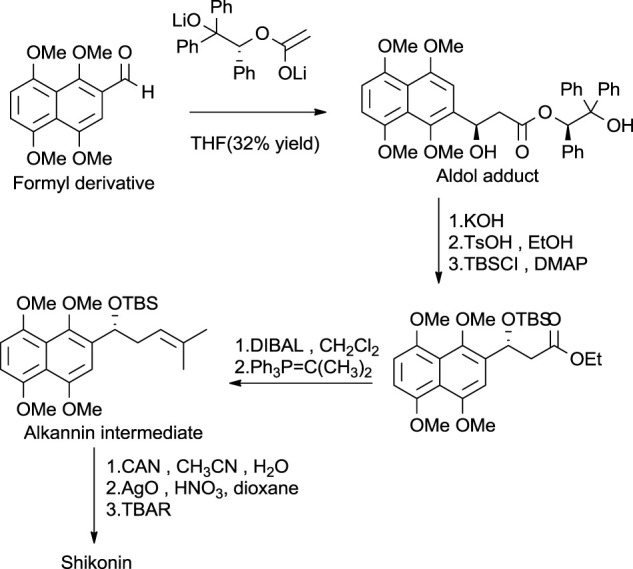
Baurer and Braun’s asymmetrical approach for shikonin synthesis (Papageorgiou et al., 1999).

Another asymmetric approach was reported by Braun and Bauer (1991) (Scheme 17), in which aldol addition takes place into the formyl derivative by using acetate enolate equivalent (Papageorgiou et al., 1999). The aldol adduct was treated in an alkali medium with TsOH (p-Toluenesulfonic acid), forming an alkannin intermediate, which on oxidation produced shikonin. As mentioned above, the synthesis of shikonin is achieved by various synthetic pathways. Continued interest in developing synthetic routes of shikonin production increases the possibility of shikonin production with safe and economic reagents.

## 6 Shikonin Derivatives—Occurrence and Stability

**FIGURE 5:**
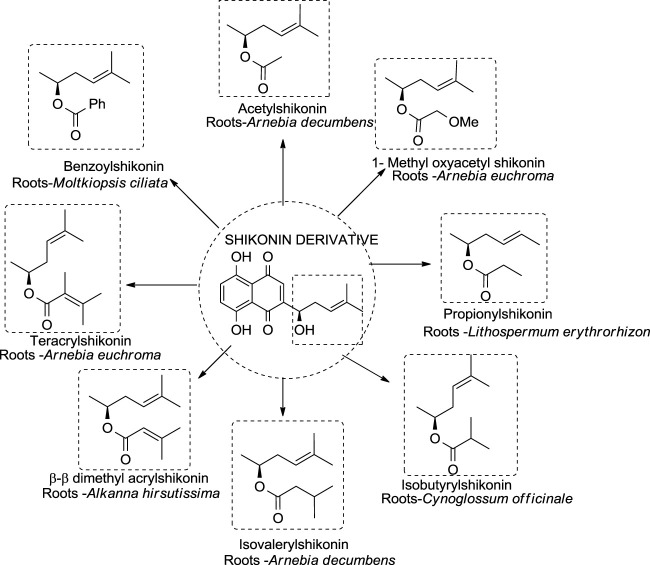
Shikonin derivatives and their occurrence (Papageorgiou et al., 2006).

Different shikonin derivatives and their occurrence in the roots of various plants are presented in Figure 5. After isolation and purification from *L. erythrorhizon,* five shikonin derivatives were isolated shikonin, deoxyshikonin, acetylshikonin, β-hydroxyisovalerylshikonin, and isobutyrylshikonin (Cho et al., 1999). The heat and light stability of these compounds was studied, and it was found that the more photodegraded or thermally degraded a compound is, the more unstable it becomes. Based on the half-life, the compounds deoxyshikonin and isobutyrylshikonin were the most thermally unstable compared to other derivatives. However, in light, all five shikonin derivatives possessed a similar half-life indicating almost similar stability (Cho et al., 1999).

## 7 Synthesis of Shikonin Derivatives

**SCHEME 18:**
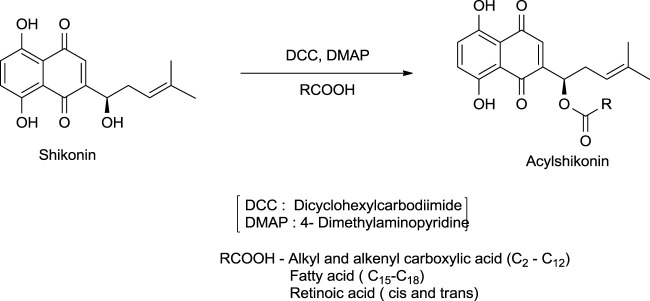
Synthesis of acylshikonin (Ahn et al., 1995).

**FIGURE 6:**
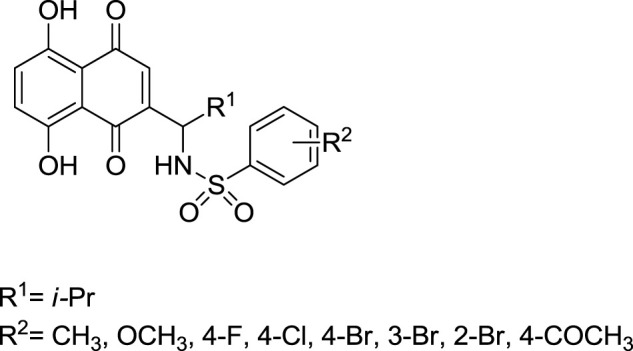
Structure of shikonin analogues (Wang et al., 2009).

**SCHEME 19:**
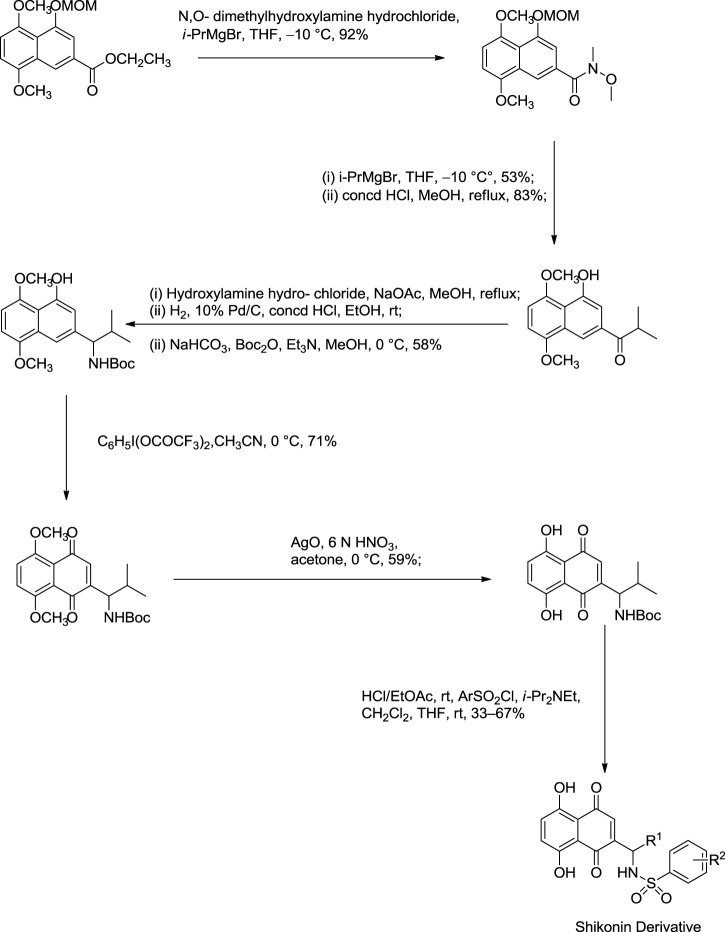
Synthesis of shikonin derivative (Wang et al., 2009).

The synthesis of acylshikonin from shikonin by reaction with DMAP (4-(dimethylamino)pyridine) in the presence of DCC (dicyclohexylcarbodiimide) has been reported (Scheme 18) (Ahn et al., 1995). The shikonin derivatives with structures shown in Figure 6 were more potent against various cancer cell lines, making the synthesis of these analogues important. Wang developed the synthesis of shikonin derivatives as shown in Scheme 19 (Wang et al., 2009).

**SCHEME 20:**
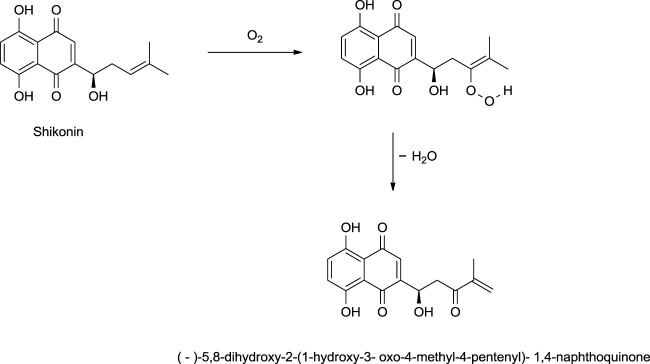
Photooxidation of shikonin (Cheng, 1995).

**SCHEME 21:**
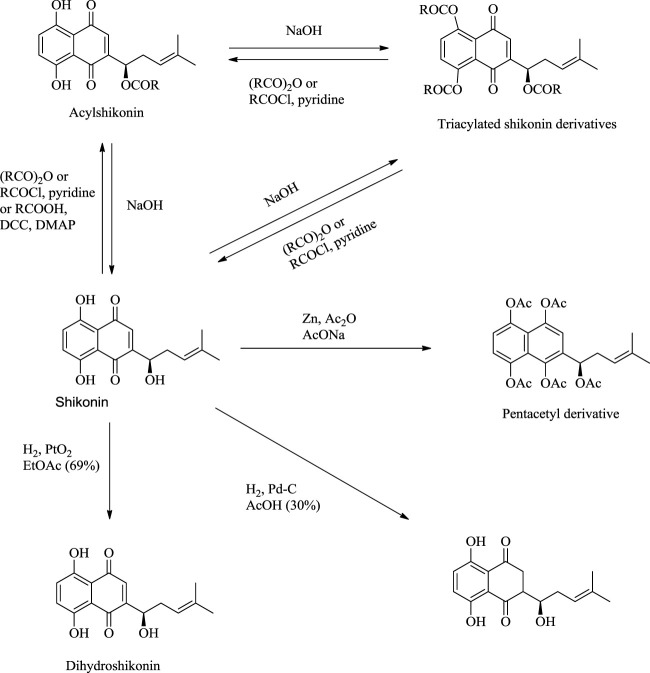
Reactions of shikonin (Papageorgiou et al., 1999).

Shikonin, on exposure to light and air, produces (-)-5,8-dihydroxy-2-(1-hydroxy-3-oxo-4-methyl-4-pentenyl)-1,4-naphthoquinone as a significant product *via* a mechanism given in Scheme 20 (Cheng, 1995). The reduction of shikonin with Zn or Na_2_SO_4_ (sodium sulfate) produced a tetrahydroxynaphthalene derivative, which is highly air-sensitive and was caged as a pentaacetyl derivative (Scheme 21) (Papageorgiou et al., 1999). Hydrogenation of shikonin with Pd catalysis reduces both the quinine and alkene groups. When PtO_2_ (platinum (IV) oxide) was used as a catalyst, only the alkene group was reduced (Scheme 21) (Papageorgiou et al., 1999).

## 8. Pharmacology of Shikonin and its Derivatives

Shikonin and its derivatives have been very well known for their beneficial effects on humans from ancient times. Major pharmacological applications of shikonin and its derivatives are discussed.

### 8.1 Anti-Inflammatory Effects

**SCHEME 22:**
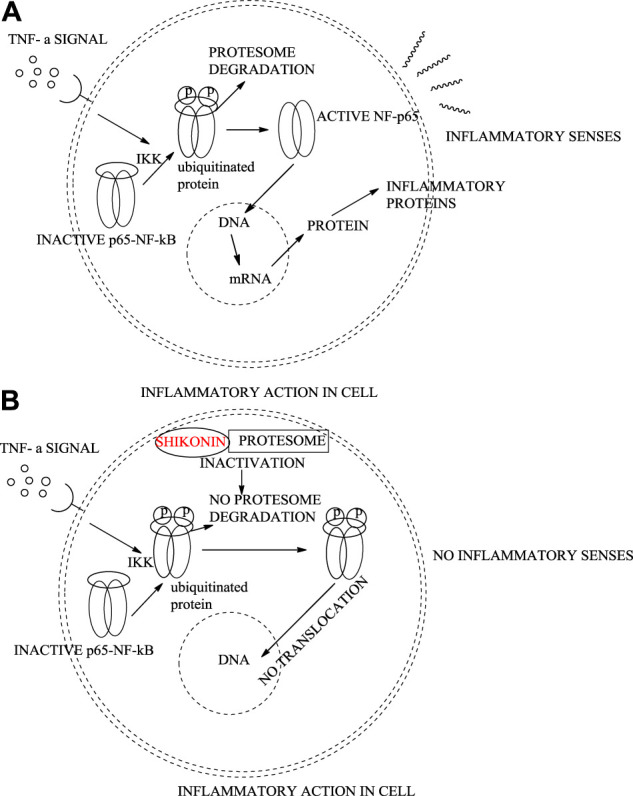
Regular inflammatory activity in an epithelial cell **(A)**, shikonin inhibiting protesome showing anti-inflammatory activity **(B)**.

Shikonin extracted from the roots of *L. erythrorhizon* and *A. euchroma* exhibits anti-inflammatory effects (Tanaka et al., 1986). Shikonin can inhibit inflammation *in vivo* by inhibiting the release of the mediator TNF-α (tumour necrosis factor α, an inflammatory mediator) in rat macrophage cells (Han et al., 2021). It also inhibits lipopolysaccharides (LPS)-mediated-NF-κB (nuclear factor kappa-light-chain-enhancer of activated B cells) translocation from the cytoplasm to the nucleus. In addition, the proteasome in macrophage cells is also inhibited by shikonin, causing cell death (Scheme 22) (Lu et al., 2011).

Shikonin at four mg/kg is as efficient as dexamethasone (2.5 mg/kg), which is known as the most effective anti-inflammatory agent. LPS-mediated TNFα, released in macrophage cultures, is completely inhibited by 4 μM of shikonin. Shikonin also induces apoptosis partially, which is better anti-inflammatory therapy than inducing necrosis of inflammatory cells. Shikonin at 1 μM dose induces a several-fold increase of ubiquitinated proteins, which induce proteasome inhibition (Lu et al., 2011).

**FIGURE 7:**
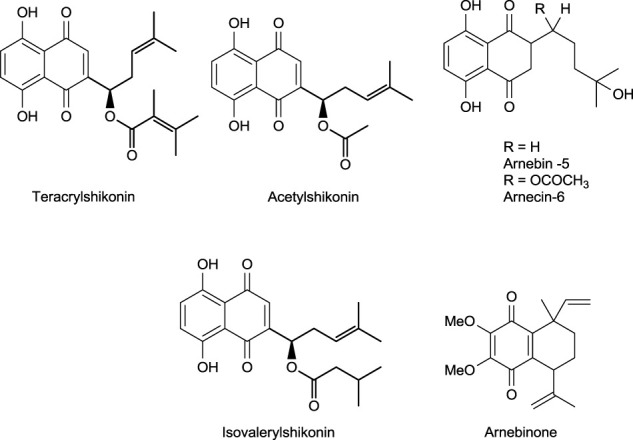
Shikonin derivatives with potent anti-inflammatory activity (Singh et al., 2003).

In another study (Kundakovic et al., 2006), *O. leptanhtha* roots containing three different derivatives of shikonin exhibited anti-inflammatory effects. Using the Carrageenan-induced rat paw edema tests, anti-inflammatory effects of shikonin derivatives were found (Kundakovic et al., 2006), with acetyl shikonin exhibiting better anti-inflammatory effects (Figure 7).

Shikonin inhibits allergic reactions *via* mobilization of Ca^2+^ and as an antagonist of Mas-related G-protein-coupled receptor X2 (Wang et al., 2020). One study shows shikonin efficiency in treating neuroinflammation by reducing D-galactose, which causes memory impairment and neuron damage other than neuroinflammation (Zhong et al., 2020). *A. hispidissima* roots extracted with ethanol generated many shikonin derivatives, which were found to have anti-inflammatory effects due to certain compounds such as arnebinone and acetyl shikonin (Figure 7). At a 5 mg/kg dose for 8 h, shikonin showed 27.17 (moderate) and 37.94% inhibition of inflammation, respectively (Singh et al., 2003). Although shikonin was shown to be an effective anti-inflammatory agent, it exhibits different action pathway mechanisms at various doses. In a recent study, data showed the potential of shikonin (50 mg/kg dose) in treating sepsis-induced and acetaminophen-induced acute liver injury causing inflammation (Guo et al., 2019). The *in vivo* study shows that shikonin regulates microRNA-140–5p/toll-like receptor 4, which results in healing lung injury (Zhang et al., 2020).

### 8.2 Wound Healing Effects

Various studies revealed that shikonin has considerable potential for wound healing and treating different scars (Fan et al., 2019). The wound healing process includes different steps like cell proliferation, inflammation, matrix deposition, and tissue remodelling. During damaged tissues, repair, collagens, fibronectin, and transforming growth factor-*β*1 are synthesized, which play an essential role in wound healing (Sidhu et al., 1999). A natural product isolated from *Arnebia Nobilis Rech. f.*, arnebin-1, increases the formation of transforming growth factor *β*-1, collagens, and fibronectin, thereby accelerating the process of wound healing (Sidhu et al., 1999). The derivatives of shikonin *viz.* acetylshikonin, isovalerylshikonin, and *β-β*, dimethyl acrylshikonin extracted from *A. tinctoria* are found to possess sound wound-healing effects (Papageorgiou et al., 1999). Histoplastin red ointment, very well known for its wound-healing benefits, contains alkannin esters (Papageorgiou et al., 1999). The bark of *O. echioides* roots containing shikonin also exhibits wound healing properties (Nikita et al., 2015). The water extract of *L. erythrorhizon* has been used for its wound healing properties by promoting the migration and proliferation of dermal fibroblasts with increased lipid synthesis (Kim et al., 2012). An advanced study showed the effectiveness of a novel electrospun carrier of Alkannin/Shikonin for wound healing. The polymeric nanofibers composed of cellulose acetate (CA) or poly (ε-caprolactone) (PCL) containing a different ratio of Alkanin/Shikonin showed potential antibacterial properties, specifically dimeric A/S, wherein shikonin showing toxicity at 500 nM and 1 μM and alkannin at 1 μM (Arampatzis et al., 2021). Another polymer, poly [(R)-3-hydroxybutyric acid] (PHB) fibre mat carrying Shikonin/Alkannin, also shows potential antimicrobial activity *via* cell proliferation (Arampatzis et al., 2021). These sightings indicate that entrenching Shikonin/Alkannin and their derivatives into various nanofibers and other nano-carriers might be a beneficial and efficient drug delivery. Further, these natural products imbedded nanofibers and nano-carriers may also serve as a potential contender for biomedical applications of bioactive natural products in the area of skin and bone tissue engineering.

### 8.3 Antitumoral and Anti-Cancer Effects

**FIGURE 8:**
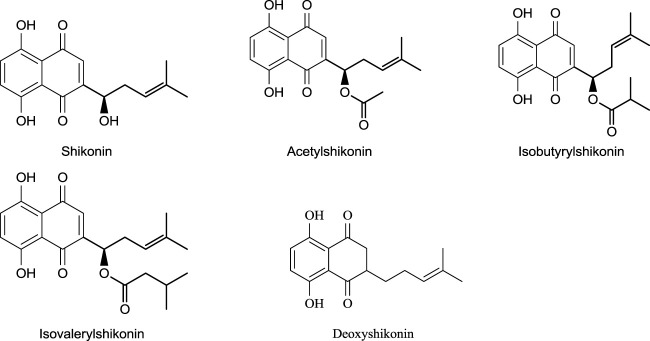
Shikonin derivatives with potent anticancer activity (Rajasekar et al., 2012).

*L. erythrorhizon* roots extract has been used as a traditional medicine in the Asian continent for its effect on skin cancers (Rajasekar et al., 2012). Shikonin and isobutyrylshikonin have potent activity against oral cancer cells, and isobutyrylshikonin is a more effective anti-cancer agent than shikonin in an *in vitro* study (Park et al., 2020). Moreover, shikonin possesses a differential regulation mechanism against hepatocellular carcinoma, showing variable sensitivity for shikonin (Yang et al., 2021). Anticancer activities, including cell growth, cell cycle, apoptosis, and tumour regulating protein, were analyzed against melanoma cells to evaluate *in vitro* and *in vivo* effects of shikonin (Liu et al., 2020; Cao et al., 2020; Wu et al., 2004). Shikonin hindered the growth of melanoma cells *in vivo* after 21 days. Under *in vitro* conditions, shikonin promotes the regulation of apoptotic and inhibits the promotion of anti-apoptotic (Rajasekar et al., 2012). A 10 mg/kg dose of shikonin for 21 days reduced tumour cell growth by 43% and 36% weight. Shikonin derivatives also are effective for anti-cancer activity (Rajasekar et al., 2012) and shown in Figure 8 **.**

**SCHEME 23:**
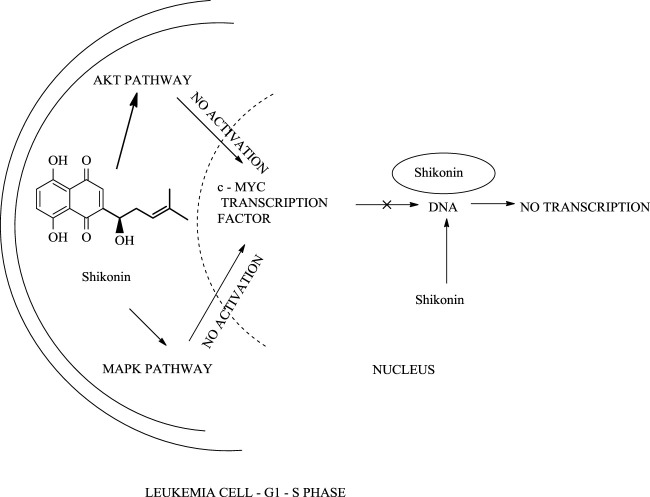
The action of shikonin on leukaemia cell.

In another study, shikonin slows the growth of cervical cancer cells by inhibiting tumour growth (50% inhibition) and hence promotes tumour cell death (Ma et al., 2020). When HeLa cells (immortal cell line taken from Henrietta Lacks) were treated with a 40 μmol/L dose of shikonin, Hela cells undergo apoptotic activity, including a series of changes, for example, change in cell shape, membrane, and DNA fragmentation, which block the transition of HeLa cell from the G1 (growth phase 1) phase to the S (synthesis phase) phase in the cell cycle. Shikonin’s anti-cancer activity against HeLa cells can be increased by combining melatonin with shikonin (Li et al., 2020). Shikonin and its derivatives were effective against leukaemia cell lines, U937 (cell line model) **(** Scheme 23) (Zhao et al., 2015). Recent studies depict the implementation of shikonin as a potential therapy against breast cancer (Chen et al., 2019).

**SCHEME 24:**
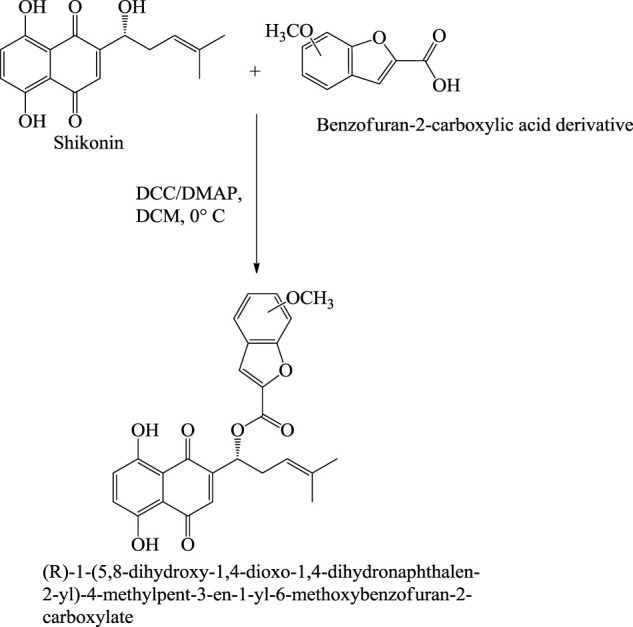
Synthesis of shikonin-benzo[b]furan derivatives (Shao et al., 2020).

With the advancement of science and technology, researchers from all over the world have been focusing on the synergistic detoxification effects of potent drug molecules in combination or association with other components such as drug-metal nanoparticles, drug-carbon dots, and drug-related derivatives. These types of synergistic detoxification methodologies have emerged rapidly in recent years, particularly in cancer and other deteriorative ailments’ treatment Advancement in cancer treatment studies show the potential of shikonin-AgNPs (silver nanoparticles) nanoparticles against A549 cells (human lung carcinoma cell line) (Fayez et al., 2020). Shikonin used in the synthesis of AgNPs reduced the hazards related to toxic chemicals, and together they possess inhibitory and proliferation activity against A549 cells (Fayez et al., 2020). The IC50 of shikonin-AgNPs determined by MTT assay after 24 h was 2.4 ± 0.11 μg/ml (Fayez et al., 2020). Further, Yu-Ying Shao et al. designed a series of novel shikonin-benzo [b]furan derivatives (Scheme 24), which were found to possess anticancer activity against HT29 (human colorectal adenocarcinoma) cell lines better than shikonin and with low cytotoxicity against non-cancer cells (Shao et al., 2020). The shikonin-benzo [b]furan derivatives induce cell apoptosis and cell cycle arrest and inhibit tubulin polymerization—the derivative acts as a competitive binder to tubulin against colchicine. The IC50 value of the derivative against HT29 was recorded as 0.18 μM, which supports the anti-cancer property of the derivatives (Shao et al., 2020). Another study shows the mechanism of the anti-leukaemia effect of shikonin as it binds and inhibits the expression of c-MYC and affects the phosphorylation of AKT, ERK1/2, and SAPK/JNK (Zhao et al., 2015); Zhao et al. (2015) evaluated shikonin and its fourteen derivatives against U937 leukaemia cells for their anti-leukaemia potential. Out of fourteen, only four derivatives (β,β-dimethylacrylshikonin, isovalerylshikonin, 2-methylbutyrylshikonin and isobutyrylshikonin) were found to be more active as compared to shikonin. AnnexinV-PI studies showed that shikonins tempted apoptosis. G1/S check point regulation of Cell cycle and c-MYC transcription factor that plays a significant role in cell cycle proliferation and regulation was noted as the most usually down-regulated mechanisms upon curing with shikonins in mRNA microarray hybridizations. Further, DNA-binding and western blotting assays inhibited transcriptional activity and c-MYC expression by shikonins. The retardation of c-MYC expression was allied with deregulated AKT (serine/threonine-specific protein kinase), ERK (Extracellular signal-regulated kinase), MAPK (mitogen-activated protein kinase) and JNK (c-Jun N-terminal kinase) activity, representing their engrossment in shikonin-triggered c-MYC deactivation. Molecular docking investigations presented that shikonin, along with its derivatives, binds to the similar DNA-binding area of c-MYC. This result displayed that shikonins bind to c-MYC. The effect of shikonin on U937 cells was also confirmed in another leukemia cell lines (Molt4, Jurkat, multidrug-resistant CEM/ADR5000 and CCRF-CEM), where shikonin also known to prevent c-MYC expression and affected phosphorylation of SAPK/JNK, ERK1/2 and AKT. Overall, the c-MYC inhibition and other related pathways signifies a substantial pathway of shikonin and its derivatives to enlighten their anti-leukemic potential (Zhao et al., 2015). Table 5 represents some anti-tumor and anti-cancer studies of shikonin.

**TABLE 5:** Recent anti-tumor and anti-cancer studies.

Shikonin action	Cell or tumor model	Mechanism	References
Inhibiting cell proliferation	MCF-7 BC cells	G_0_/G_1_ arrest of breast cancer multiplying cells.	Yang et al. (2019)
	HCC cells	Inhibiting pyruvate kinase type M2	Liu et al. (2020b)
	Human melanoma A375 and A2058 cells	Inhibition on STAT3 signaling	Cao et al. (2020)
Apoptosis	Melanoma cells (B16F10)	Up- regulation of Bax expression	Rajasekar et al. (2012)
	Mice models (C57BL/6)	Down-regulation of Bcl-2	
	COLO 205 cells	Down-regulation of Bcl-2 and Bcl-XL up-regulation of p27, p53, and Bad	Pulley and Czakó (2004)
	HeLa Cells	SIRT3/SOD2-AKT pathway inhibition via reduction in SIRT3/SOD2 expression and SOD2 activity	Li et al. (2020)
	Human lung cancer cells (A549)	Upregulation of p53 expression	Yeh et al. (2015)
	Glioma cells (Hs683 cells)	Regulating endoplasmic reticulum via stress-mediated tumor apoptosis which target caspase-3 and Bax/Bak-induced mitochondrial outer membrane permeabilization.	Ma et al. (2020)

### 8.4 Antiprotozoal Activity of Shikonin

Ali et al. (2011) evaluated various distinct naphthoquinones for their antileishmanial activities against *Leishmania major* by an *in-vitro* method. These distinct naphthoquinones were assessed for their cytotoxic potential against BMMΦ (bone marrow-derived macrophages). The study revealed that the naphthoquinones members of the Shikonin/alkannin group displayed noticeable antileishmanial potential with less toxicity against BMMΦ at various tested concentrations. Furthermore, Shikonin and alkannin were found to have potent antiprotozoal potential when tested against *Leishmania major* by inhibiting the growth of extracellular parasites with IC_50_ values of 0.5–6 μM (Ali et al., 2011). Shikonin and alkannin’s structure/activity relationship study against *Leishmania major* showed exciting results. The addition of methoxy group or methyl at the C2 position of the parent structure of naphthoquinone increased the antiprotozoal potential.

**FIGURE 9:**
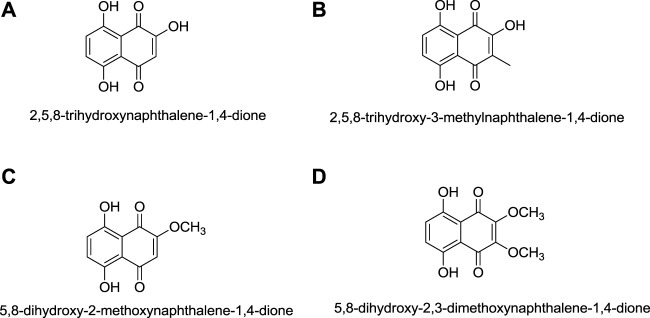
Structure/Activity relationship of 1,4- naphthoquinone.

In contrast, hydroxyl group at the same position decreased the activity of shikonin and alkannin (Ali et al., 2011). Moreover, hydroxylation at the C5 position and dihydroxy group substitution at C5 and C8 positions help to increase the antiprotozoal potential. These results revealed that the mode of action of various distinct naphthoquinones is deceptively governed by the substitution pattern of different functionality (Figure 9; Ali et al., 2011).

### 8.5 Other Pharmacological Uses

Shikonin and its derivatives have been mainly known and used for their anticancer, antitumor, anti-inflammatory and wound healing potential. Owing to this, only the pharmacological properties of shikonin and its derivatives have been discussed in the present article. However, Shikonin and its derivatives also show other pharmacological properties such as anti-adenovirus, anti-parasite, hypoglycemia, antioxidant, anti-bacterial, anti-obesity, anti-ear oedema, anti-diabetic, anti-fungal, anti-osteoporosis (Table 6). Furthermore, shikonin and its derivatives showed a wide range of antioxidant potential against various free radicals and reactive nitrogen and oxygen species. The antioxidant potential of shikonin and its derivatives is mainly attributed to the presence of a phenolic ring which helps to provide proton and free-electron to neutralize the various reactive species (Charan Raja et al., 2016; Hao et al., 2020; Nasrollahzadeh Sabet et al., 2020). The pharmacological study of shikonin and its derivatives show various applications against various diseases. These studies reveal the mechanism of action and efficiency of shikonin and its derivatives, which will help to understand the shikonin action against various other diseases which are yet to be discovered.

**TABLE 6:** Pharmacological uses of shikonin and its derivatives.

Pharmacological uses	Recent studies	References
Anti-adenovirus	Adenovirus type 3 (AdV3) growth is hindered by 0.0156–1 mM dose of shikonin *in vitro* with a virus reduction rate of 23.8%–69.1%.	Gao et al. (2011)
Anti-parasite	Shikonin derivative acetylshikonin kills intercellular parasite *Leishmania donovani* by promoting interleukin IL—10 and IL-12, nitric oxide, and reactive oxygen species (ROS).	Charan Raja et al. (2016)
Hypoglycemia	Hypoglycemia is a condition where the blood sugar level is dropped down. Shikonin was found to be effective in this state by increasing the glucose uptake by myocytes (muscle cells) and adipocytes (fat cells) and having very less effect on protein tyrosine phosphorylation in the cells.	Nigorikawa et al. (2006)
Antioxidant	Cigarette smoke contains a high amount of peroxyl radicals (measured by ESR spectrometry). Peroxy radicals are the main reactive oxygen species (ROS). Shikonin being a better ROS scavenger exerts the scavenging activity against peroxyl radicals, therefore shikonin is an effective antioxidant.	Nishizawa et al. (2005), Hao et al. (2020), Nasrollahzadeh Sabet et al. (2020)
Anti-bacterial	Due to the presence of three hydroxyl groups in shikonin, it acts as free ion scavengers, and the bacteria have their membrane made up of ion gradient hence destroy bacteria membrane and possess high antibacterial activity. Moreover, the ring structure of the shikonin acts as protoplasm toxic and inhibits protein synthesis in bacteria which kills the bacteria. Shikonin has also shown anti-biofilm activity against *Listeria monocytogenes.*	Li et al. (2021)
Anti-obesity	Shikonin (concentration—1.1 mM) hinder the synthesis of fat droplet and triglyceride in adipocytes cells (3T3-L1) by inhibiting the expression of a gene which are included in lipid metabolism. Shikonin inhibits the regulation of adipocytes cells expressions and as a result, downregulates lipid metabolizing enzymes. Finally reducing fat accumulation.	Jang et al. (2008)
Anti-ear oedema	50–1.0 mg dose of shikonin reduces the oedema, 70% cyclooxygenase-2 expression, and 100% of inducible NO synthase under *in vivo* by decreasing the translocation of protein kinase, phosphorylation, and activation of ERK. It also hinders the binding of NF-kB-DNA. Hence lessen the inflammatory effect of ear oedema	Andújar et al. (2010)
Anti-diabetic	Shikonin and acetylshikonin offers potential against type II diabetes by showing agonism activities on free fatty acid receptor 4, which is novel target for treating type II diabetes.	Xu et al. (2021)
Anti-Fungal	Shikonin is an antifungal agent against *Candida albicans* and *Aspergillus terreus* by inhibiting fungal growth and killing the biofilms by regulating various genes which inhibit hyphae formation.	Yan et al. (2019), Shishodia and Shankar (2020)
Prevent Osteoporosis	Shikonin promotes bone marrow mesenchymal stem cells differentiating into osteoblasts via β-catenin signaling pathway and inhibit the formation of osteoclasts.	Zhou et al. (2021)

## 9 Toxicity of Shikonin

**FIGURE 10:**
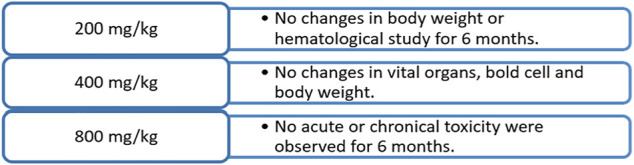
Toxicology study of shikonin on adult Wistar rats (Su et al., 2014).

**FIGURE 11:**
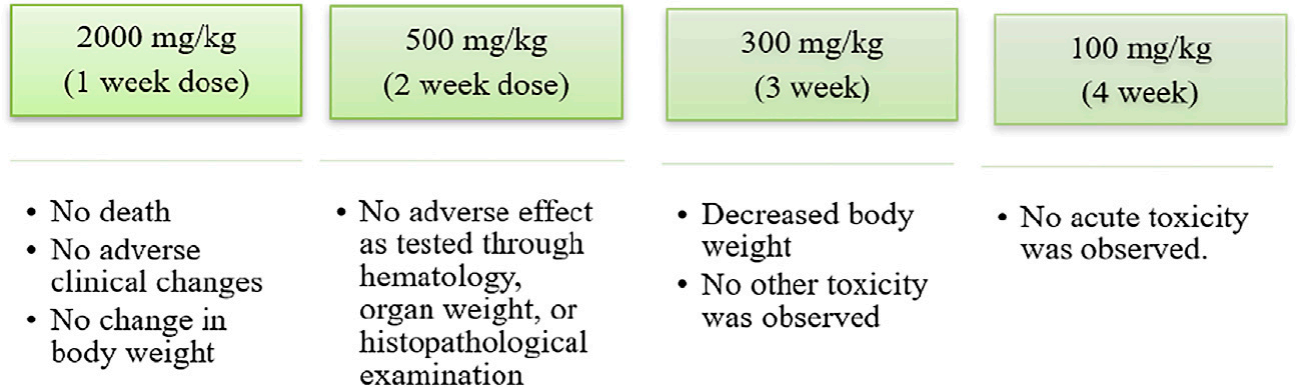
Toxicology study of shikonin on beagle dog (Nam et al., 2015).

Shikonin and its various derivatives have various applications in pharmacology, from being traditional Chinese herbs to modern medicines. Moreover, shikonin and its derivative impart less toxicity to the tissues to which they are applied. For example, adult Wistar rats treated with a microemulsion solution of shikonin at 200, 400, and 800 mg/kg for 90–180 days (Su et al., 2014) were examined constantly with haematological and biochemical analyses. No acute or chronic toxicity was found, and hence shikonin was concluded to be safe (Figure 10) (Su et al., 2014). In another toxicology study conducted on Beagle dogs, it was shown that a weekly oral dose of shikonin for 4 weeks had no or minor adverse effects (Figure 11) (Nam et al., 2015).

Another *in-vivo* study of shikonin against renal fibrosis in a mouse model of unilateral ureteral obstruction (UUO) targeted various glycolytic enzymes. It inhibited renal fibrosis, but weight loss in mice was also a side effect (Wei et al., 2019). The shikonin was administrated to the mouse model as an intraperitoneal injection with a 5 mg/kg/day dosage on alternate days. Moreover, when compared to dichloroacetate, shikonin was more toxic and less potent in inhibiting fibrosis (Wei et al., 2019). Finally, a recent study of skin-sensitization agents in food and cosmetics presents interesting data using a local lymph node assay with an elicitation phase (Yamashita et al., 2018). Shikonin was a potential skin-sensitization compound even at a low concentration (0.05%) (Yamashita et al., 2018).

Recent studies suggest the toxicity of shikonin on liver tissue of humans and rats as an inhibitor of uridine 5′-diphosphate-glucuronosyltransferase (Cheng et al., 2019). (Figat et al., 2021) showed the cytotoxicity of shikonin against regular cell lines V79 was high (Table 7). The shikonin derivative, acetylshikonin is less cytotoxic but possesses antigenotoxicity against cyclophosphamide-induced genotoxicity (Figat et al., 2021). The three different cytotoxic assays show a low value of EC_50_ (half-maximal effective concentration) for shikonin than acetylshikonin. This result demonstrates that shikonin has higher cytotoxicity against regular cell lines than acetylshikonin (Figat et al., 2021).

**TABLE 7:** Cytotoxicity of shikonin and acetylshikonin against V79 cell lines (Figat et al., 2021).

Sr. No.	Cytotoxicity assay	EC50	
		Shikonin	Acetylshikonin
1.	LDH Assay	0.18 mg/L	0.49 mg/L
2.	MTT Assay	0.40 mg/L	1.16 mg/L
3.	NRU Assay	0.60 mg/L	1.32 mg/L

The *in-vitro* study of shikonin against cancer cells shows potent cytotoxicity against 15 cancer cell lines *via* mitochondrial dysfunction and leads to cell apoptosis in the cancer cell (Wiench et al., 2012). Interestingly, in the same study, shikonin also showed systemic toxicity against regular cell lines (Wiench et al., 2012). Another *in-vitro* study shows shikonin inducing suicidal erythrocyte death, i.e., eryptosis, by stimulating Ca^2+^ entry, forming ceramide, resulting in phosphatidylserine translocation and shrinkage of human erythrocytes cells (Lupescu et al., 2014). The *in-vitro* study of the inhibition risk of shikonin on cytochrome P450 in mammals shows mixed and a competitive inhibitor of CYP1A2, CYP2B6, CYP2C9, CYP2D6, CYP3A4, and CYP2E1 (Tang et al., 2017). This could cause high drug-drug or food-drug interaction toxicity as cytochrome P450 enzymes are essential for drug metabolism in mammals (Tang et al., 2017).

A difference can be observed between *in vivo* and *in vitro* studies of toxicity; *in vivo* studies, less toxicity is observed than *in vitro*. The administration mode of shikonin also played a vital role in determining toxicity, as evidenced in the above-mentioned *in vivo* studies. The acute toxicity data related to the mode of administration revealed that when administrated orally (in mice), the LD50 was noted to be >1 g/kg. When administrated intraperitoneally (in mice), the LD50 was observed to be 20 mg/kg, whereas when given intravenously (in rabbits), the LD50 was 16 mg/kg (https://cdn.caymanchem.com/cdn/msds/14751m.pdf). These findings revealed that the oral administration of shikonin produces no or very little toxicity. However, when administrated as intraperitoneal and intravenous injections, specific toxicity has been observed in the tested animal. Hence, direct application or injection dose produces toxicity as a high amount of shikonin targets the desired tissue. This may be due to the different rates of absorption of drugs and exposure to different environmental conditions when given by diverse routes. Apart from this, various other factors such as metabolism, toxic kinetics, and experimental uncertainty may also affect the toxicity level of any drug when given by intravenous or intraperitoneal, or oral routes. All these factors contribute to the actual bioavailability of the drug at the target site. Although shikonin can be metabolized in the body when administrated via any route, when given by oral route, the first-pass metabolism occurs along with a lower absorption rate at the intestine, which is not observed in other routes. This will result in the decreased bioavailability of shikonin, and hence toxicity in the oral route is more minor compared to the other mode of administration. Owing to the above studies, considerable attention should be given to the issues related to the safety of shikonin in combination with other drugs *via* associated metabolic enzymes *in vivo*. Therefore, further research in this area is needed to understand the mechanism and level of toxicity of shikonin and its derivatives.

## 10 Patents Filed and Clinical Trials of Shikonin and Its Derivatives

Owing to the wide range of pharmacological and medicinal applications of shikonins, several patents have been filed related to shikonin production from various natural sources and their usefulness in the field, as mentioned above. The details of various patents filed are provided in Table 8. In addition, due to the high pharmacological benefits of the shikonin various clinical trials have been carried out to evaluate their efficacy: some key ones (source- https://clinicaltrials.gov/) are listed in Table 9. From ancient times, *Lithospermum erythrorhizon* extract or shikonin has been extensively used to treat ulcers, wounds and burns. Further, shikonin based pharmaceutical formulations with wound healing potential have been in the market for the distant past. Despite this, only a few clinical studies have been registered involving shikonin and its derivatives. Karayannopoulou et al. (2011) compare the shikonin derived human ointment with Ringer’s lactate solution on dogs for its wound healing potential. The result revealed that the scores of collagen formation, tissue perfusion, and angiogenesis of wounds cured with shikonin derived ointment in the healing course were flagrantly higher than Ringer’s lactate solution cured wounds (Karayannopoulou et al., 2011). These clinical studies are vital to validate the effectiveness of shikonin and its derivatives in treating various diseases. This will tremendously accelerate and promote the clinical alteration of shikonin-based drugs in the future.

**TABLE 8:** Patents filed on shikonin and its derivatives.

Sr.No.	Patent name	Scientist name	Patent number and year
1.	Medicine containing shikonin compound as an active component (Feixin 2008)	Wang Feixin	CN100370976C, 2008
2.	Pharmaceutical composition comprising shikonin derivatives from *Lithospermum erythrorhizon* for treating or preventing diabetes mellitus (Hwang et al., 2010)	Ji Ho Park, Sun Yeou Kim, Tong Ho Kang Eun Ju Hwang, and Chul Hoon Kang	US20100093852A1, 2010
3.	Use of alkannin in preparing medicine for treating tumor disease (Xun and Jianping 2006)	Hu xun, and Fangjianping	CN1579378A, 2006
4.	Lithospermum and application of its active ingredient in preparing medicament for treating tumors stem cell (Xùn 2012)	Hu xun	CN101194920B, 2012
5.	Method for producing complex of shikonin compound and β-1,3–1,6-glucan (Nagasaki et al., 2018)	Ken Nagasaki, Taizo Taniguchi, Mariko Takenokuchi, Suzuki Toshio, Masanori Yanagida	JP6322784B2, 2018
6.	Application of 5,8-dyhydroxyl-2-(1-acetyl-4-methyl-3-pentenyl)-1,4-naphthoquinone diketone to preparation of medicines for resisting diabetes (Liu Chaochun 2012)	Liu Chaochun, and Zhu Banghao	CN102526016A, 2012
7.	High-optical-purity alkannin and akannin naphthazarin nuclear parent hydroxyl methylation carbonyl oxime derivative and preparation method (Li Shaoshun 2013)	Li Shaoshun, Wang Rubing and Zhang Xu	CN103130680A, 2013
8.	Preparation method of high optical purity shikonin and alkannin, and derivatives (Shaoshun and Rubing 2012)	Lishao shun and Wang rubing	CN102399139A, 2012
9.	Application of shikonin in medicine for treating acute leukemia (Zhang et al., 2014)	Zhang Bo, Chen Na, Zheng Qiusheng, Chen Hongmei and Wang Xiaoqin	CN103720679A, 2014
10.	Method for extracting shikonin from lithospermum (Liuhua 2011)	Su liu Hua	CN101973864A, 2011
11.	Application of shikonin in preparing medicine for inducing apoptosis (Weidong 2007)	Hu Xun and Han Weidong	CN1931152A, 2007
12.	Coloring method of the shikonin to wool fabric (Ming et al., 2017)	Lu ming, Lilanqian liu, Xin liu, Ping liuyiping, Fan tao and Zhaozhenyun Zhou jing	CN106758344A, 2017

**TABLE 9:** Clinical trials registered on clinicaltrials.gov related to shikonin and shikonin producing species (source- https://clinicaltrials.gov/and Sun et al., 2022).

Sr.No.	Study title	Drug name	Conditions	Locations	Identifier
1.	Academia sinica investigator award 2010	Shikonin	Breast cancer	Taipei Medical University — WanFang hospital Taipei, Taiwan	NCT01287468
2.	The role of pyruvate kinase M2 in growth, invasion and drug resistance in human urothelial carcinoma	Shikonin	Bladder urothelial carcinoma	Department of urology, National Taiwan University hospital Taipei, Taiwan	NCT01968928
3.	A Series of N-of-1 trials of traditional chinese medicine based on bayesian method	Radix lithospermi	Bronchiectasis	Yueyang Hospital of Integrated Traditional Chinese and Western Medicine, Shanghai University of Traditional Chinese Medicine Shanghai, China	NCT04601792
4.	Evaluating the effects of traditional chinese medicine by N-of-1 trials	Radix lithospermi	Bronchiectasis	Yueyang Hospital of Integrated Traditional Chinese and Western Medicine, Shanghai University of Traditional Chinese Medicine Shanghai, Shanghai, China	NCT03147443
5.	Effectiveness of Qufeng Shengshi Fang on treatment of allergic rhinitis.	Radix lithospermi	Rhinitis, allergic, perennial	Peking Union Medical College Hospital, Traditional Chinese Medicine Department Beijing, Beijing, China	NCT02653339
6.	Study of tumor-shrinking decoction (TSD) to treat symptomatic uterine fibroids	Lithospermum erythrorhizon	Leiomyoma	School of Chinese Medicine, University of Hong Kong Hong Kong, China	NCT02189083

## 11 Nano Delivery System for Shikonin

The literature revealed that shikonin has low solubility in an aqueous solution and requires chemical stability. Owing to this, shikonin has low *in-vivo* biological activity (Cao et al., 2018; Lin et al., 2018). Further, the haphazard toxicity of shikonin also resulted in the unease of pharmaceutical practitioners (Zhang et al., 2020). Therefore, various unified approaches and methods are essential to address these pressing and vital problems. Recently, various nano-functional-based delivery systems such as liposomes, microemulsions, micelles, nanoparticles, and nanomaterials have shown immense potential in enhancing the *in-vivo* bioavailability of shikonin and its enhanced biological stability, prolonged *in-vivo* residence, reduced adverse reactions and increased tissue distribution.

Furthermore, the RFC11 and shikonin containing liposomes prepared by the lipid preparation technique have been exploited for inducing necrotic ptosis. Concurrently, the *in-vivo* healing potential of prepared liposomes was assessed from the orthotopic and subcutaneous colon carcinoma model of CT26 murine colon adenocarcinoma cells in mice. The result demonstrated that the liposomes tempted supreme tumour regression in the two models (Agarwalla and Banerjee, 2016). Furthermore, Shikonin containing liposomes synthesized from phospholipids, cholesterol and soybean phospholipids have been reported to suggestively increase the impedance rate of angiogenesis of ICR and tumour migration in mice and noticeably reduce toxicity. The results showed that shikonin containing liposomes might be an effective medication for clinical application in cancer patients (Xia et al., 2013). Cancer immunotherapy is one of the best strategies for the cure of cancer.

Nevertheless, immunosuppression tumour microenvironment and immune tolerance are the key obstacles in Cancer immunotherapy. Coincidentally, a multifunctional nanoparticle delivery system that helps co-deliver shikonin and PD-L1 knockdown siRNA help to overcome this uneasy situation. Furthermore, subsequent studies have established that the siRNA could efficiently inhibit PD-L1 and help to improve the response of cytotoxic T lymphocytes to tumour cells and repolarize tumour-allied macrophages from the M2 to M1 subtype states. Thus, these nano-based delivery system offers an excellent possibility for cancer immunotherapy (Li et al., 2020).

Presently, most traditional administration systems fail to target specific infected organs. Thus, the administrated drug is distributed evenly in the body. This high dose is required to initiate healing effects *in-vivo*, which results in increased toxicity and various other side effects. Therefore, the target-specific delivery is a substantial measure to improve drug efficiency and reduce toxicity. The study revealed that the shikonin-silver nanoparticles established promising targeting performance to the lung as the maximum lung radioactivity accumulation of 31.20% ± 1.5% and the radiolabeling yield of 97% ± 2.8% ID/g after injection (Fayez et al., 2020). Microemulsion also provides selective biological delivery properties for water-insoluble drugs, similar to liposomes. Furthermore, it was reported that biofunctionalized microemulsion (prepared from AS1411 aptamer/hyaluronic acid) co-loaded with shikonin and docetaxel could penetrate the blood-brain barrier and accumulate effectively in the brains of tumour-bearing (orthotopic luciferase-transfected U87 glioma) nude mice. Thus, these results offer a probable strategy for anti-glioma treatment (Wang et al., 2019).

In recent years, there have been very few adverse reports related to different nanodrug delivery systems. Nowadays, these nano delivery systems are growing gradually due to their various advantages regarding accurate and organ-specific drug delivery, which results in increased bioavailability and lower toxicity. Thus, these nano delivery systems can allow shikonin or other bioactive secondary metabolites with similar chemical and physical properties to exert their complete therapeutic potential for the cure of various diseases *in-vivo* along with lesser side effects.

## 12 Future Aspects

The beneficial effects of shikonin on human health and various other purposes have been well exploited. Researchers have analyzed biosynthetic and other pathways of shikonin production from various plant sources other than the roots of *L. erythrorhizon* (Yazaki, 2017). Genetically engineered biosynthetic pathways of shikonin have been utilized to improve shikonin yield and production.

There is a clear danger of overexploitation, leading to the extinction of shikonin-producing plants. We are responsible for protecting the shikonin-producing plants; thus, various other plants are now being discovered that produce shikonin or its derivatives. Also, we have to focus on new efficient chemical and biotechnological sound synthetic methods to produce a high yield of shikonin in the future. Apart from these, more efficient delivery systems based on the biocompatible and biodegradable natural and synthetic polymers for target specific delivery of shikonin and its derivatives should be explored soon.

Recent studies have demonstrated shikonin benefits in food preservation and checking the pH of foods. The secondary metabolite: shikonin can be a better alternative as a food preservative than synthetic preservatives. Further, the freshness of meats can also be identified with the help of shikonin, as it can help check the pH of the meats. Advances in these studies can lead to a new area of shikonin uses. Research studies show the potential of shikonin and its derivatives against various diseases, and many are yet to be discovered. However, shikonin’s toxicity and its derivatives need a more thorough investigation of safety and regulation before shikonin can be considered a potent drug.

## 13 Conclusion

Medicinal plants have been widely used since ancient times to cure various ailments, and today their demand has been grown on a large scale to meet industrial demand. Shikonin-producing plants have been a good source of medicine for treating various diseases with very few adverse effects. Various methods developed to isolate shikonin, and its derivatives from shikonin-producing plants have been discussed in this article. The use of genetically engineered biosynthetic pathways will help attain high yields of shikonin and a large number of other derivatives for eventual extraction. It is expected that laboratory synthesis of shikonin and its derivatives will lessen the exploitation of shikonin-producing plants and can become an alternative method to meet shikonin demand in the market. Shikonin has countless medicinal benefits and has been used to treat several deteriorative diseases. This article has also discussed the pathways or modes of action through which shikonin acts upon any disease. Shikonin has proven to be an efficient and less toxic potent bioactive phytoconstituent to maintain the quality of life for humans. However, various pharmacological applications, clinical studies and toxicological investigations of shikonins and their derivatives have been discussed in the present article. Still, various domains have not been explored yet. Thus, soon the studies should be carried out to explore the new synthesis or production pathways, methods of efficient delivery, and *in-vitro* and *in-vivo* pharmacology assays, not only to define and clarify the molecular targets for shikonin and its derivatives but also to evaluate their clinical potential.

## Glossary

3T3-L1: Adipocytes cells
4HB: 4-hydroxybenzoic acid
AdV3: Adenovirus type 3
Akt: Protein kinase B
BAP: 6-benzylaminopurine
Bcl-2: B-cell lymphoma 2
BAX: Bcl-2-associated X protein
CAN: Ammonium cerium(IV) nitrate
CDI: 1,1′-Carbonyldiimidazole
CoA: Coenzyme A
DCC: Dicyclohexylcarbodiimide
DDQ: 2,3-Dichloro-5,6-dicyano-1,4-benzoquinone
DMAP: 4-(dimethylamino)pyridine
DNA: Deoxyribonucleic acid
DSHs: Deoxyshikonin hydroxylases
EC50: Half maximal effective concentration
ER: Endoplasmic reticulum
Erk: Extracellular Receptor Kinase
ESR: Electron paramagnetic resonance
FW: Fresh Weight
G1: Growth phase 1
GBA: 3-geranyl-4-hydroxybenzoate
GPP: Geranyl diphosphate/geranyl pyrophosphate
HCC: Hepatocellular carcinoma
HeLa cells: Immortal cell line taken from Henrietta Lacks
HIV: Human immunodeficiency Virus
HMG: 3-hydroxy-3-methylglutaryl
HMG-CoA: 3-hydroxy-3-methylglutaryl-coenzyme A
HMGR: 3-hydroxy-3-methylglutaryl reductase
IBA: Indole-3-butyric acid
IL: Interleukin
IUPAC: International Union of Pure and Applied Chemistry
LDH: Lactate dehydrogenase
LeSAT1: shikonin O-acyltransferase
LeAAT1: alkannin O-acyltransferase
LPS: Lipopolysaccharides
LS: Linsmaier and Skoog medium
MAPK: Mitogen-activated protein kinases
MCF-1: Michigan Cancer Foundation-7
MOM: Methoxymethyl ether
MTT: 3-(4,5-dimethylthiazol-2-yl)-2,5-diphenyltetrazolium bromide
NF-Κb: Nuclear factor kappa-light-chain-enhancer of activated B cells
NRU: Neutral Red Uptake
OTMS: Octadecyltrimethoxysilane
Pd: Palladium
pH: Potential of Hydrogen ion
PHB: *p*-hydroxybenzoic acid
Py: Pyridine
ROS: Reactive oxygen species
S: Synthesis phase
SIRT3: Sirtuin 3
SOD2/Akt: Superoxide dismutase 2- Protein kinase B
STAT3: Signal transducer and activator of transcription 3
TBAR: Thiobarbituric acid reactive substances
TBSCl: *tert*-Butyldimethylchlorosilane
TBSO: *tert*-Butyldimethylsilyl ethers
THF: Tetrahydrofuran
TNF-α: Tumor necrosis factor α
TsOH: p-Toluenesulfonic acid
U937: Cell line model
